# Divergent composition and transposon-silencing activity of small RNAs in mammalian oocytes

**DOI:** 10.1186/s13059-024-03214-w

**Published:** 2024-03-26

**Authors:** Li Hou, Wei Liu, Hongdao Zhang, Ronghong Li, Miao Liu, Huijuan Shi, Ligang Wu

**Affiliations:** 1 Key Laboratory of RNA Science and Engineering, Shanghai Institute of Biochemistry and Cell Biology, Center for Excellence in Molecular Cell Science Chinese Academy of Sciences, University of Chinese Academy of Sciences, Shanghai, 200031 China; 2 Shanghai-MOST Key Laboratory of Health and Disease Genomics, NHC Key Lab of Reproduction Regulation, Shanghai Institute for Biomedical and Pharmaceutical Technologies, Shanghai, 200032 China

**Keywords:** piRNA, PIWI, Transposon, Endo-siRNA, Oocytes

## Abstract

**Background:**

Small RNAs are essential for germ cell development and fertilization. However, fundamental questions remain, such as the level of conservation in small RNA composition between species and whether small RNAs control transposable elements in mammalian oocytes.

**Results:**

Here, we use high-throughput sequencing to profile small RNAs and poly(A)-bearing long RNAs in oocytes of 12 representative vertebrate species (including 11 mammals). The results show that miRNAs are generally expressed in the oocytes of each representative species (although at low levels), whereas endo-siRNAs are specific to mice. Notably, piRNAs are predominant in oocytes of all species (except mice) and vary widely in length. We find PIWIL3-associated piRNAs are widespread in mammals and generally lack 3′-2′-O-methylation. Additionally, sequence identity is low between homologous piRNAs in different species, even among those present in syntenic piRNA clusters. Despite the species-specific divergence, piRNAs retain the capacity to silence younger TE subfamilies in oocytes.

**Conclusions:**

Collectively, our findings illustrate a high level of diversity in the small RNA populations of mammalian oocytes. Furthermore, we identify sequence features related to conserved roles of small RNAs in silencing TEs, providing a large-scale reference for future in-depth study of small RNA functions in oocytes.

**Supplementary Information:**

The online version contains supplementary material available at 10.1186/s13059-024-03214-w.

## Background

There are three types of small non-coding RNAs found in animal germlines, including microRNAs (miRNAs), endogenous small interfering RNAs (endo-siRNAs), and PIWI-interacting RNAs (piRNAs) [1]. MiRNAs and endo-siRNAs are ~ 22-nt long and bind to Argonaute (AGO) proteins to form RNA-induced silencing complex (RISC) [2]. miRNAs are processed from hairpin-containing primary transcripts (pri-miRNA) by Drosha and Dicer sequentially [3], whereas endo-siRNAs are produced from endogenous, long double-stranded RNAs by Dicer cleavage [4, 5]. Depletion of Dicer or AGO2 in mouse oocytes results in meiotic arrest accompanied by severe spindle and chromosomal segregation defects, extensive transcripts misregulation, and female sterility [6–8]. By contrast, inactivation of Drosha and DGCR8 has no obvious effect on normal oocyte maturation [9, 10]. Together with several other studies [11, 12], those observations in mouse oocytes suggest that Dicer-dependent endo-siRNAs are the main players controlling oocyte development and female fertility, while miRNA function is globally suppressed. However, the presence and function of endo-siRNAs and miRNAs in other mammals remain elusive.

PiRNAs are another type of small RNAs which are reportedly found mainly in germ-line cells and bind to PIWI proteins to form the piRNA-induced silencing complex (piRISC) [4, 13–16]. PiRNAs are generated from discrete genomic loci, called piRNA clusters, which harbor remnants and nested fragments of transposable elements (TEs) that are unable to transpose [17]. TEs are mobile elements that mobilize within their host genome and threat genome integrity. The piRNA pathway serves as an effective defense system against TEs employed by nearly all animals [18]. Once TE sequences are inserted into a piRNA cluster, corresponding piRNAs are produced from transcripts of this cluster to silence the invading TEs, in a proposed mechanism termed the “trap model” [19, 20]. PIWIs and piRNAs are widely expressed in the gametes of frogs, flies, mice, bovines, and pigs, and their appropriate expression is essential for successful reproduction [13, 14, 21–25]. In *Drosophila* and zebrafish, the disruption of any PIWIs or piRNA pathways causes sterility in both females and males [26, 27]. The three PIWIs found in mouse testis, including PIWIL1 (MIWI), PIWIL2 (MILI), and PIWIL4 (MIWI2), are expressed at different developmental stages and loss of individual Piwi proteins leads to a spermatogenic arrest, whereas female mice remain fertile [13, 28–30]. The *Piwil3* gene is absent in the mouse and rat genome, but is encoded in almost all other sequenced mammalian genomes. PIWIL3 has been detected in bovine, monkey, and human oocytes and shown to be associated with oocyte short piRNAs (os-piRNAs), which are shorter than canonical piRNA families and lack the 3′-2′-O-methylation in [16, 31]. Notably, recent studies in golden hamsters have revealed that os-piRNAs are also highly expressed in the female germline and that disruption of the piRNA pathway impairs both male and female fertility [32–34], thus establishing the indispensability of piRNAs in mammalian oocytes.

Previous efforts have highlighted the diversity of small RNA composition and function among human, mouse, monkey, and golden hamster oocytes [5, 16, 31, 35, 36]. However, except for a few species, the expression profiles of small RNAs in other mammalian oocytes remain largely uncharacterized. In this study, we analyze the small RNA profiles in oocytes of 12 animal species, including human, monkey, mouse, rat, golden hamster, Chinese hamster, rabbit, guinea pig, dog, pig, goat, and zebrafish, which reveals high diversity in the composition of small RNA populations of mammalian oocytes. Moreover, we identify syntenically conserved and species-specific piRNA clusters, highlighting the relationship between TEs and piRNAs. Our work provides a valuable reference and contextual basis for further investigation of small RNA diversity and function in evolutionary events and heritable human diseases.

## Results

### Oocyte small RNA profiles vary widely among mammals

**Fig. 1:**
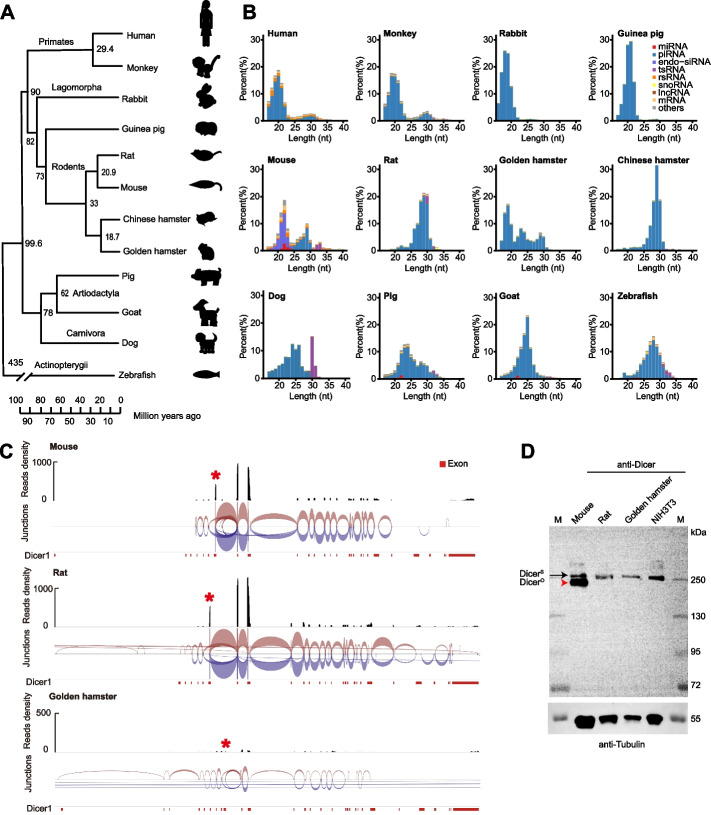
Small RNA composition and length distribution in oocytes from 12 representative vertebrate species. **A** Phylogenetic tree showing relationships between the 12 vertebrate species used in this study. The numbers on the tree indicate estimated divergence time in millions of years ago. **B** Small RNA composition and length distribution in oocytes of the 12 representative vertebrate species. **C** Sashimi plot of splice junctions in Dicer expressed in oocytes of mouse, rat, and golden hamster. The red asterisk indicates the RNA-seq signal of the MT-C element. IGV was used to compute the junction track from alignment data [38]. Junctions from the + strand are colored red and extend above the center line. Junctions from the − strand are blue and extend below the center line. Arc height and thickness are proportional to the depth of read coverage. **D** Detection of endogenous Dicer1 proteins in mouse, rat, and golden hamster MII oocytes via western blot. The black arrow indicates Dicer^S^ and the red arrowhead indicates Dicer^O^. Dicer was detected using 100 mouse, 75 rat, and 44 golden hamster oocytes. Approximately 1 μg of total protein was used for NIH3T3 Dicer detection. Dicer^S^, somatic Dicer1; Dicer^O^, oocyte Dicer1; M, marker

We sequenced both small RNAs and mRNAs in oocytes from 12 animal species including 11 mammals (human, monkey, rabbit, guinea pig, mouse, rat, golden hamster, Chinese hamster, dog, pig, goat), representing ~ 81 million years of evolution within the eutherians (Fig. 1A). All oocytes were collected at the germinal vesicle (GV) or MII stage (Additional file 1: Fig. S1). For each species, 3–5 individual oocytes were sequenced. On average, approximately 11 million reads were obtained per sample (i.e., individual oocyte) for small RNA libraries and 5 million reads per sample were obtained for poly(A)-bearing long RNAs libraries; all libraries were composed of more than 80% high quality reads (Additional file 2: Table S1-S2). The cDNA insertions ranging from 17 to 40 nt in length were used to identify small RNAs. The small RNA profiles of mouse, golden hamster, monkey, and human oocytes were consistent with previous studies [5, 16, 35, 36] supporting the reliability of our sequencing data. The piRNA candidates, which contained a strong 1 U bias at the 5′ end and enrichment of 10-nt overlap of piRNA pairs (Additional file 1: Fig. S2A-B), had varying length distributions between animal models and represented the most dominant RNA species, ranging from 80 to 98%, in the oocytes of 11 mammals, except mice (Fig. 1B, Additional file 1: Fig. S2C). In mouse oocytes, piRNAs comprised a relatively low proportion (25%), while endo-siRNAs were the predominantly expressed small RNAs (32% of total small RNAs) (Additional file 1: Fig. S2C). Intriguingly, endo-siRNAs were undetectable in the other 10 mammalian oocytes. MiRNAs were generally present in the oocytes of all species analyzed here, accounting for 0.05 to 6% of total small RNA reads, while tsRNAs represented 0.44–11.0% of the total small RNAs. These results suggest that, in contrast to the conserved patterns of predominant miRNA expression in somatic cells [37], small RNA composition in the oocytes is highly variable between species, potentially reflecting the specialization of small RNA functions in female germ cells.

### Endo-siRNAs are uniquely expressed in mouse oocytes

Endo-siRNAs are known to function as an “innate immune” system to defend against viruses in plants and flies [39, 40], and to silence TEs in plants, flies, and *C. elegans* [19]. Our results showed that endo-siRNAs comprised a small RNA class exclusively in mouse oocytes among the 12 species (Fig. 1B). Analysis of miRNA and siRNA pathway gene expression in our oocyte RNA-seq data revealed globally low expression levels of mRNAs across species (Additional file 1: Fig. S3, Additional file 2: Table S3). A previous study found an N-terminally truncated isoform of Dicer (Dicer^O^) resulting from a mouse transcript C (MT-C) retrotransposon insertion which could process dsRNAs more efficiently than wild-type Dicer, thus generating abundant endo-siRNAs in mouse oocytes [41]. This MT-C retrotransposon is rodent-specific and Dicer^O^ is reportedly expressed in both mouse and rat oocytes [41]. We confirmed that the sequence of MT-C element was present in reads of the *Dicer* gene in our RNA-seq data from mouse, rat, and golden hamster oocytes (Fig. 1C). Intriguingly, Western blot data indicated that Dicer^O^ protein was the dominant isoform, compared to wild type, in mouse oocytes, but was undetectable in rat and golden hamster oocytes (Fig. 1D), suggesting posttranscriptional regulation of Dicer^O^ protein expression. Notably, a portion of the incorporated MT-C elements occupied the full 5′ untranslated region (UTR) and the alternative first exon (AltE) of Dicer^O^ transcripts [41]. However, multiple mutations and indels were identified in the rat MT-C elements compared to the mouse MT-C sequence (Additional file 1: Fig. S4A), which shared 84% sequence identity with rat MT-C elements (Additional file 1: Fig. S4B). These sequence variations could potentially result in the diminished Dicer^O^ expression observed in rat oocytes compared to that in mice. We then constructed four plasmids expressing a GFP reporter fused to the 5′ UTR of Dicer^O^ transcripts and the AltE from mouse and rats, in all four possible combinations (i.e., mouse 5′ UTR plus mouse AltE; mouse 5′ UTR plus rat AltE, etc.; Additional file 1: Fig. S4C). GFP expression varied among the four combinations, with fusion to the rat 5′ UTR resulting in the lowest expression levels (Additional file 1: Fig. S4D-E), suggesting that the MT-C elements inserted in the 5′ UTR of rat Dicer^O^, along with other possible factors, might reduce or abolish its expression. Consistent with the absence of Dicer^O^ expression, no endo-siRNAs were detected in either rat or golden hamster oocytes in these small RNA sequencing data (Fig. 1B). These findings led us to hypothesize that, in contrast with mice, the *Dicer* MT-C retrotransposon insertion in rat and golden hamster may not result in the production of Dicer^O^, which could further explain the lack of endo-siRNAs in rat and golden hamster oocytes. Altogether, these data show that both Dicer^O^ and endo-siRNAs are likely mouse-specific among mammalian oocytes.

### Low miRNA expression is conserved in mammalian oocytes

**Fig. 2:**
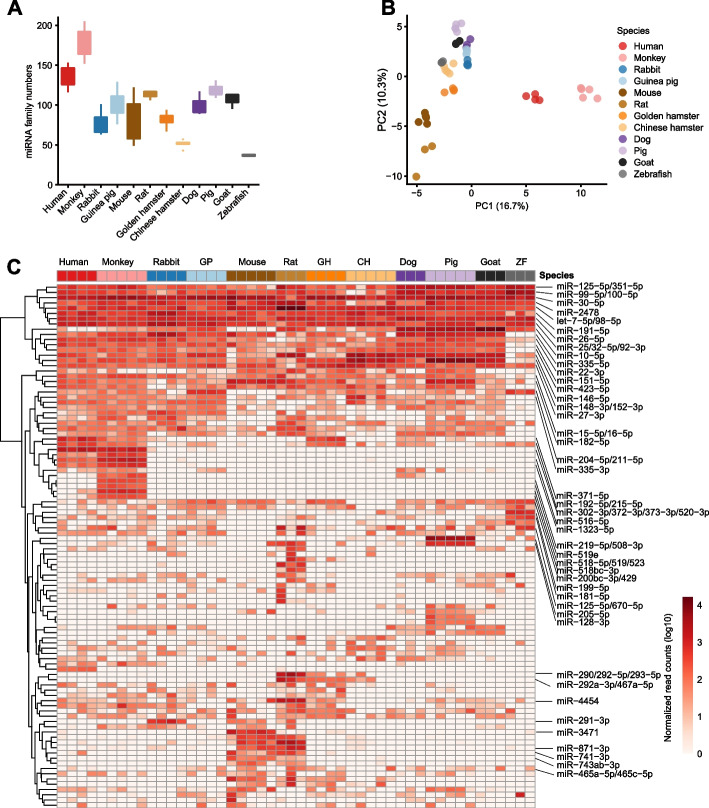
miRNAs are ubiquitously expressed at low levels in mammalian oocytes. **A** The number of miRNA families detected in oocytes. **B** Principal component analysis (PCA) of oocyte miRNAs in the 12 representative species (total miRNA families were used for PCA analysis). **C** Heatmap of the 100 most abundant miRNA families in oocytes. Hierarchical clustering was used to generate a heatmap (method = ‘complete’). GP, guinea pig; GH, golden hamster; CH, Chinese hamster; ZF, zebrafish

We then examined the miRNA profiles in the 12 species and found that total miRNA accounted for less than 6% of total small RNAs in oocytes. We detected an average of 100 miRNA families in each species (Fig. 2A, Additional file 2: Table S4), and principal component analysis (PCA) showed obvious clustering of samples from the same species, but little overlap between species, suggesting that overall miRNA profiles are distinct among these 12 species (Fig. 2B). Notably, most of the highly expressed miRNAs, including miR-125-5p/351-5p, miR-99-5p/100-5p, miR-30-5p, miR-2478, and let-7-5p/98-5p, were conserved among the 12 vertebrates (Fig. 2C), implying that they are likely functional in oocytes. Some species-specific miRNAs were highly expressed in oocytes, such as miR-518bc-3p, miR-518-5p/519/523, and miR-519e, which were only expressed in monkey oocytes, and miR-290/292-5p/293-5p, miR-292a-3p/467a-5p, which were uniquely detected in rat oocytes (Fig. 2C). In rabbit oocytes, miR-291-3p expression was elevated to more than tenfold that of other species. The presence of highly expressed, non-conserved miRNAs suggested species-specific regulatory roles for these miRNAs in oocytes. Although miRNAs were previously shown to be dispensable for mouse oogenesis [9, 10], the genes related to miRNA production and function were relatively highly conserved across species (Additional file 1: Fig. S3), strongly suggesting that miRNAs could play an important role in mammalian oogenesis and early embryonic development. Moreover, mouse oocytes express extremely high levels of endo-siRNAs, which also require several proteins involved in miRNA processing and function [42]. These results support the possibility that mice may not be a sufficiently representative model for studying miRNA functions in oocytes.

### PIWIL3-associated piRNAs are widespread in mammalian oocytes

**Fig. 3:**
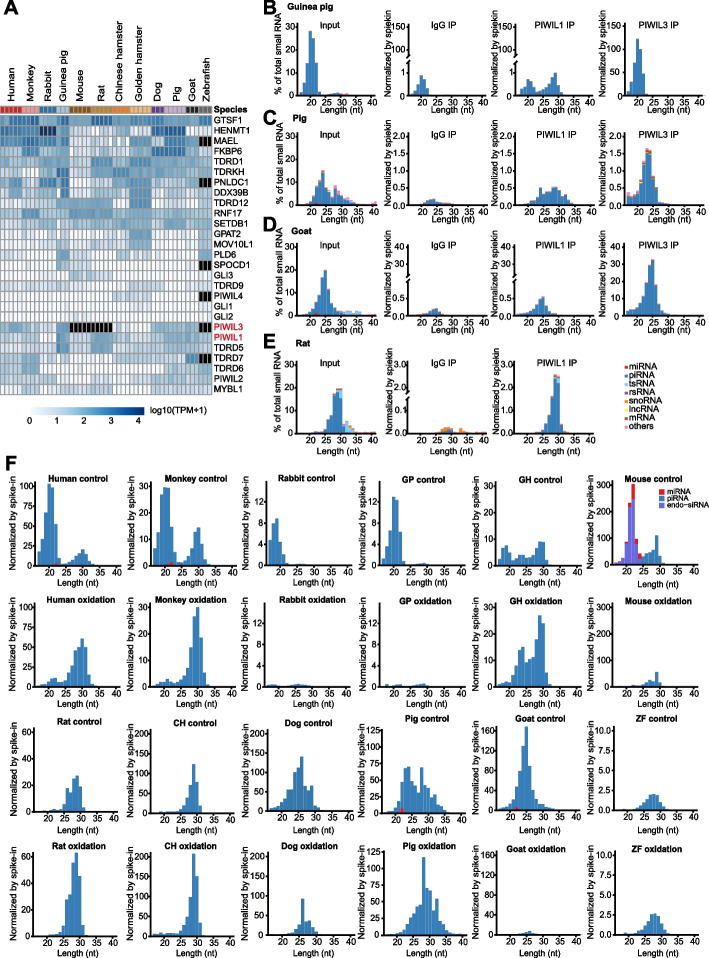
RNA immunoprecipitation sequencing of PIWI proteins and detection of 3′-2′-*O*-methylation in oocyte small RNAs. **A** Expression of piRNA pathway-related genes in oocytes from the 12 representative vertebrate species. Hierarchical clustering was used for to generate a heatmap (method = ‘complete’). Black indicates that a species did not have the corresponding gene in its genome. **B**–**D** PIWIL1 and PIWIL3 RNA immunoprecipitation analysis in oocytes from guinea pig (**B**), pig (**C**), and goat (**D**). IgG IP was used as the negative control. The *Y*-axis scale compares the amount of small RNAs between PIWIL immunoprecipitation and IgG controls within species. **E** PIWIL1 RNA immunoprecipitation analysis in rat oocytes. IgG IP was used as the negative control. *Y*-axis scale compares the amount of small RNAs between PIWIL immunoprecipitation and the IgG control within species. **F** Small RNA composition in oocyte samples treated with NaIO4 (oxidation) or in untreated samples (control). GP, guinea pig; GH, golden hamster; CH, Chinese hamster. *Y*-axis scales are used for within-species comparisons of small RNA levels with or without NaIO4 treatment

Analysis of piRNA biogenesis- and function-related genes in our oocyte RNA-seq data, such as *Pld6*, *Mov10l1,* and *Pnldc1,* indicated that these genes were expressed only at low levels, whereas *Henmt1* was highly expressed in the large majority of species examined here, especially rabbits (Fig. 3A, Additional file 2: Table S5). While *Piwil1* and *Piwil2* were expressed in almost all species, *Piwil4,* by contrast, was only expressed at low level in golden hamster and Chinese hamster. *Piwil3* expression was observed in most species, except mouse and rat, the genomes of which lack *Piwil3* genes. Previous studies have revealed that long piRNAs bind to PIWIL1, while os-piRNAs bind to PIWIL3 in human and golden hamster oocytes [16, 33]. To classify piRNAs according to binding partners in other mammalian oocytes, we generated species-specific PIWIL3-antibodies for guinea pig, goat, and pig (Additional file 1: Fig. S5A-C), and PIWIL1-antibodies for guinea pig, goat, pig, and rat (Additional file 1: Fig. S5D-G), then performed IP assays in oocytes of each respective species. Sequencing of small RNAs associated with PIWIL1 and PIWIL3 showed that 17–22-nt piRNA candidates in guinea pigs (Fig. 3B), 17–26-nt piRNA candidates in pigs (Fig. 3C), and 17–27-nt piRNA candidates in goats (Fig. 3D) all bound to PIWIL3, thus indicating that these were os-piRNAs. Alternatively, the longer candidate piRNAs bound to PIWIL1 in guinea pig and pig oocytes (Fig. 3B, C). In rat oocytes, all candidate piRNAs were relatively long and bound to PIWIL1 (Fig. 3E), which was consistent with the dominant expression of PIWIL1 and the absence of *PIWIL3* in the rat genome.

2′-O-Methyl is a typical modification at the 3′ termini of long piRNAs, but not os-piRNAs, in human, bovine, and golden hamster oocytes [16, 31, 36]. To determine the methylation status of oocyte piRNA 3′ termini in other mammals, we conducted sodium periodate (NaIO4) oxidation treatment of total oocyte RNAs in all 12 species and compared the resulting small RNA populations with that in untreated samples. In oocytes of all species, the vast majority of long piRNAs resisted NaIO4 oxidation, indicating methylation at their 3′ termini. In contrast, nearly all oocyte os-piRNAs were not modified at their 3′ termini (Fig. 3F), suggesting that os-piRNAs typically lacked 3′-2′-O-methylation. In light of these findings, it was reasonable to conclude that these oxidation-sensitive piRNA candidates were indeed os-piRNAs, including 18–22-nt piRNA candidates in monkey oocytes, 18–20-nt piRNA candidates in rabbit oocytes, and most 20–27-nt piRNA candidates in dog oocytes. Notably, among these nine mammalian oocytes that express *Piwil3*, eight expressed os-piRNAs, except Chinese hamsters, which was consistent with the absence of *PIWIL3* transcripts. In the five rodents, only golden hamsters and guinea pigs expressed *Piwil3* and os-piRNAs, while rat and Chinese hamster oocytes produced high levels of *Piwil1* and long piRNAs, and mouse oocytes contained highly abundant endo-siRNAs and low levels of piRNAs (Additional file 1: Fig. S6A). These results suggest that os-piRNAs are widespread in mammalian oocytes, except for some rodents. Overall, the os-piRNAs detected here were 18 to 22 nt in length, associated with PIWIL3, lacked 3′-2′-O-methylation, accounted for 50–90% of total piRNA in oocytes (Additional file 1: Fig. S6B), and often shared the same 5′ ends with co-expressed long piRNAs (Additional file 1: Fig. S6C).

### Conservation of piRNAs and piRNA clusters

We identified piRNA clusters by examining the genomic distribution and density of piRNAs. The number of piRNA clusters in the 12 species ranged from 71 clusters in mice to 495 clusters in monkeys (Additional file 1: Fig. S7A, Additional file 2: Table S6-S17). Despite identifying numerous piRNA clusters, the top 10 most highly expressed piRNA clusters accounted for 64 to 94% of total piRNAs in the oocytes of each species examined here (Additional file 1: Fig. S7B), which was in agreement with the patterns of piRNA expression previously reported in testis [43, 44].

**Fig. 4:**
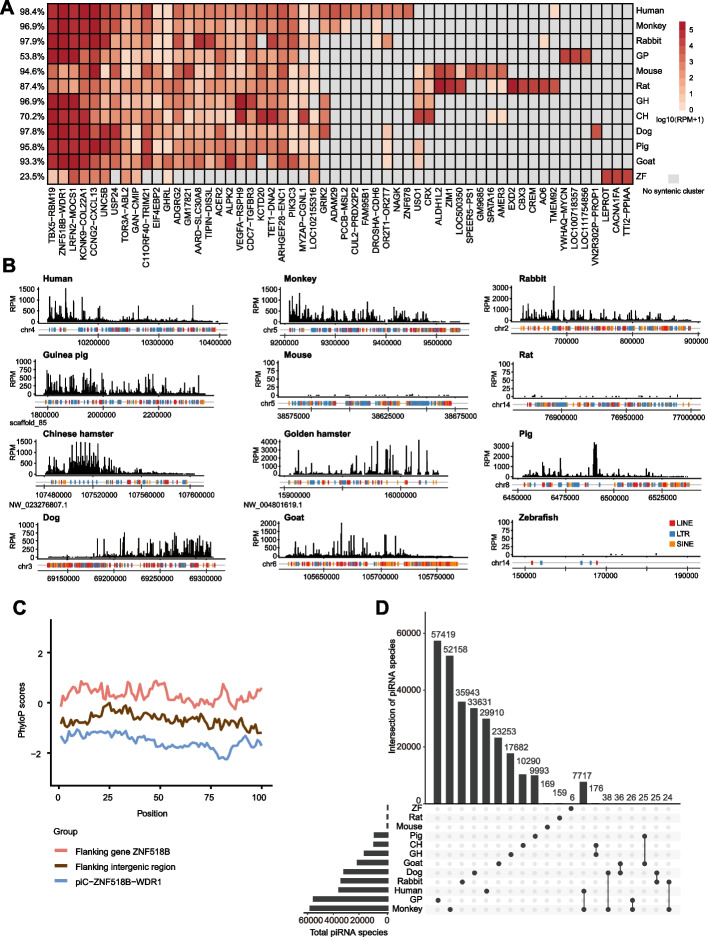
Evolutionary conservation of oocyte piRNA clusters. **A** Expression of piRNAs (quantified in RPM) in the 56 syntenic piRNA clusters in oocytes. Data are shown for piRNAs expressed in at least one species. The percentage of expressed piRNAs from the 56 piRNA clusters compared to overall oocyte piRNA expression is shown at left. Gray indicates the absence of a syntenic cluster in the indicated species. **B** Genome browser snapshots of a syntenic piRNA cluster found in several species, piC-ZNF518B-WDR1, from the 56 syntenic piRNA clusters shown in **A**. The black peaks indicate piRNA abundance in RPM. TE distributions in the corresponding genomic region are shown at the bottom. **C** The phyloP scores of the piC-ZNF518B-WDR1 of piRNA cluster, its 10 kb upstream and downstream flanking intergenic regions and cDNA sequences of adjacent protein-coding genes. The values represent -log *p*-values under a null hypothesis of neutral evolution. The sites predicted to be conserved are assigned positive scores while sites predicted to undergo accelerated evolution are assigned negative scores. **D** UpSet plot showing the identity of piRNA sequences in the piC-ZNF518B-WDR1 cluster among the 12 vertebrate species. The intersections of piRNA species are indicated by lines linking samples in the lower panel. piRNAs were classified as the same in multiple species if the piRNA length was < 23 nt and had ≤ 1 mismatch, or if the length was > 23 nt and had ≤ 2 mismatches. No mismatches were allowed in the seed region (2–8 nt). GP, guinea pig; GH, golden hamster; CH, Chinese hamster; ZF, zebrafish

To explore the evolution of piRNA clusters among species, we performed synteny analysis on a subset of piRNA clusters that were highly expressed in either most (i.e., 9 or more) of the 12 species or in only individual or a few closely related species. In total, 56 piRNA clusters were identified that generated piRNAs in at least one mammal, and these clusters explained 70–98% of total piRNA reads in 11 of the mammals, except guinea pig (~ 53%) (Fig. 4A), which may result from the low quality of genome assembly. Among the 56 piRNA clusters, 27 clusters were located in syntenic regions that were conserved across five or more species, and the expression levels of piRNAs derived from these regions varied greatly between species (Fig. 4A). The top five syntenic piRNA clusters (including piC-ZNF518B-WDR1, piC-TBX5-RBM19, piC-LRFN2-MOCS1, piC-KCNK9-COL22A1A, and piC-CCNG2-CXCL13) accounted for 40–93% of total piRNAs in each of the 11 mammals, except for rat (Additional file 1: Fig. S7C). Notably, two of these five syntenic piRNA clusters, piC-ZNF518B-WDR1 and piC-TBX5-RBM19, generated a substantial portion of piRNAs in most species (Fig. 4B, Additional file 1: Fig. S7C-D, Additional file 1: Fig. S8A).

We then examined TE composition in these two clusters and found that high TE-related piRNA levels were correlated with high TE density in the nine mammals. By contrast, in mice and rats, only a limited number of TE-related piRNAs were produced from these clusters despite the high density of TEs (Fig. 4B, Additional file 1: Fig. S8A). In order to compare conservation in the homology of genomic sequences of these two piRNA clusters and their flanking regions among species, we calculated phyloP scores for the piRNA clusters, the 10 kb upstream and downstream flanking intergenic regions, and the cDNA sequences of flanking protein-coding genes. Interestingly, the piRNA clusters had significantly lower phyloP scores than the flanking intergenic regions as well as the cDNA sequences of adjacent protein-coding genes, which suggested that these piRNA clusters were evolving more rapidly than both the flanking protein-coding and non-coding genomic regions (Fig. 4C, Additional file 1: Fig. S8B), which aligned well with reported features of testis piRNA clusters [43, 44]. The enormous diversity of piRNA sequences has been previously described in many species, including flies and mice [18]. Alignments of piRNA sequences from different species indicated very low sequence conservation (Fig. 4D, Additional file 1: Fig. S8C), which was also similar to previous studies of piRNAs in mammalian testis [44]. Notably, piRNAs from these two syntenic clusters, piC-ZNF518B-WDR1 and piC-TBX5-RBM19, corresponded to different TE subfamilies in different species (Additional file 1: Fig. S9, Additional file 1: Fig. S10). Collectively, these results suggested that, irrespective of conserved locus synteny, many piRNA clusters were rapidly evolving, and that the observed synteny was not correlated with the occurrence or expression of specific piRNAs among species.

### The piRNA levels are positively correlated with TE activity

**Fig. 5:**
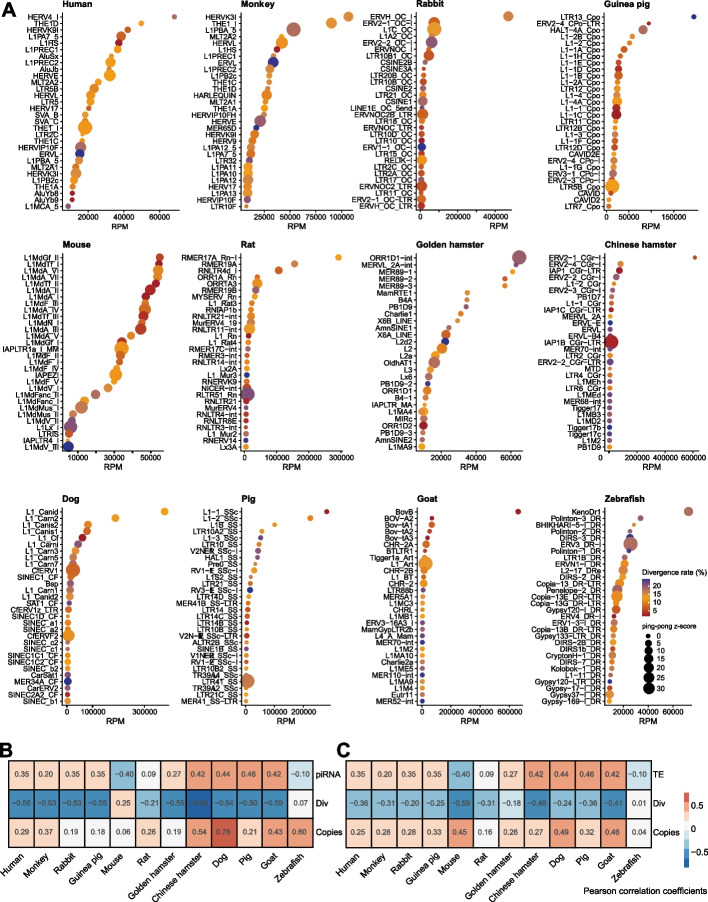
Relationship between TEs and piRNAs in mammalian oocytes. **A** Bar plot showing the 30 most abundant TE-derived piRNAs in the 12 vertebrate species. Each dot represents a TE subfamily. Color indicates the divergence rate from the consensus sequence; red indicates a lower divergence rate and blue indicates a higher divergence rate. The size of each dot indicates the *Z*-score of the ping-pong signature. **B** Heatmap of Pearson’s correlation coefficients between TE mRNA levels and three other variables: piRNA abundance, TE divergence rate, and TE copy number. TE, expressed levels of TE mRNAs; Div, average divergence of TE families; Copies, total copy number of TE families in the genome. **C** Heatmap of Pearson’s correlation coefficients between piRNA abundance and three other variables (TE mRNA levels, TE divergence rate, and TE copy number). Red indicates a positive correlation and blue indicates a negative correlation

The proportion of TEs in the genome of the 12 species ranged from 21 to 46% (Additional file 1: Fig. S11A). To investigate the relationship between piRNAs and TEs in different species, we selected the top 30 TE families with highly expressed piRNAs in each species and ranked them by piRNA abundance. The top most abundant TE-related piRNAs were expressed at substantially higher levels than other TE-related piRNAs in most species (Fig. 5A). We then integrated piRNA expression levels with their ping-pong signal, which reflects the silencing activity by PIWI-piRNAs [17, 45], and the divergence rate of each TE subfamily, which reflects their evolutionary age [46, 47]. This analysis revealed that TE families with lower divergence rates were often correlated with higher piRNA expression levels in most species (Additional file 1: Fig. S11B), meaning that younger TEs were more likely to be regulated by piRNAs. However, divergence rates were not strictly correlated with piRNA level within each species, with some highly divergent TEs still showing an abundance of related piRNAs, especially in zebrafish (Fig. 5A, Additional file 1: Fig. S11B). Notably, almost all of the low divergence TEs tended to have more abundant piRNAs than those from highly divergent families in mice and goats (Fig. 5A). Furthermore, ping-pong signal intensity varied widely within each species; universally weak ping-pong signals were identified in dogs, pigs, goats, and Chinese hamsters, while relatively stronger ping-pong signals were detected in monkeys, rabbits, and mice, potentially implying the occurrence of species-specific cleavage activity for TE transcripts by piRISCs (Fig. 5A).

To investigate whether piRNA production shared a common relationship with TE amplification across species, we explored correlations between piRNA abundance, TE mRNA levels, TE divergence rates, and TE copy numbers in individual species. This analysis showed that TE transcript abundance was correlated with increased copy number and decreased divergence rate (Fig. 5B, Additional file 1: Fig. S12), while similarly, TE-related piRNA production was correlated with higher transcript levels, higher copy number, and lower divergence rate of TEs in most mammals (Fig. 5C, Additional file 1: Fig. S12). In conclusion, actively expanding or recently expanded TE subfamilies in the mammalian oocyte genome tended to produce higher levels of piRNAs. The positive correlation between piRNA abundance and TE transposition implies that piRNAs may preferentially regulate more recently introduced TEs which potentially pose a greater threat to the host genome, which is extremely relevant in germline cells.

## Discussion

Our study illustrates the highly diverse expression of small RNAs in the oocytes of different mammals. While miRNAs are globally expressed among mammals, our findings indicate that endo-siRNAs are only present in mice. As the dominant class of small RNAs in oocytes, piRNAs vary widely in their length distributions among species. Among them, PIWIL1-piRNAs and PIWIL3-piRNAs are the two major piRNA families produced in mammalian oocytes, either alone or together, and in species-specific combinations. The 11 mammals analyzed here can be categorized into four representative groups based on their small RNA profiles. In humans, monkeys, pigs, and golden hamsters, PIWIL3-piRNAs coexist at comparable levels with PIWIL1-piRNAs. In oocytes of guinea pigs, rabbits, and goats, PIWIL3-piRNAs are the dominant piRNA subfamily; although a similar pattern is evident in dogs, these PIWIL3-piRNAs could not be validated in this study due to a lack of dog-specific PIWIL3 antibodies for immunoprecipitation. In rats and Chinese hamsters, only PIWIL1-piRNAs are expressed. In mice, PIWIL2-piRNAs coexist with endo-siRNAs [5], making mice an outlier among mammals.

Our study also suggests diverse functions for different small RNAs in gene regulation and TE repression in the oocytes of different mammals. miRNAs are mainly involved in regulating protein-coding genes. The conservation of miRNA expression in oocytes of different species may reflect their evolutionarily conserved biological functions. Intriguingly, the high levels of endo-siRNAs observed in mouse oocytes appear unique among the 11 mammals studied here, suggesting the possibility that mammals might express endo-siRNAs at generally very low levels, with mice representing an exception. A recent study revealed that endo-siRNAs bind to Ago2 to repress LTR retrotransposons in mouse oocytes, with almost all predicted targets of MILI-bound piRNAs belonging to LTR retrotransposons, and which comprise a subset of endo-siRNAs targets [48], possibly explaining the redundancy of the piRNA pathway for female fertility in mice, whereas the piRNA pathway is likely indispensable for oocyte competence in mammals lacking endo-siRNAs. PIWIL1-deficient golden hamsters produce non-functional oocytes, and PIWIL1-piRNAs may regulate both TEs and protein-coding genes [33, 34], although PIWIL3-deficient oocytes are almost indistinguishable from wild type at the transcriptome level and some of which may be fertilized and develop into pups [34]. In this study, PIWIL1-piRNAs and PIWIL3-piRNAs were found to be widely expressed in mammalian oocytes, although these two classes of piRNAs showed highly differential expression among species, implying that they are responsible for non-redundant functions in mammalian oocytes. As TEs appear to be targeted by piRNAs or endo-siRNAs in a species-specific manner, we speculate that the high diversity in small RNA composition observed in mammalian oocytes may be shaped by TEs, which show enormous diversity across species. Alternatively, the adaptive evolution of effector proteins in the piRNA pathway has been reported in previous studies [49], suggesting that they may gain some regulatory functions beyond TE repression, which could profoundly affect gamete function and embryonic development.

The piRNA pathway constitutes a defense system against TEs that threaten genomic stability in animal germline cells. The prevalent trap model proposes that piRNA clusters function in trapping TE copies that randomly transpose into these specialized genomic loci [19]. However, further study is needed to determine whether newly invading TEs preferentially insert into certain pre-existing piRNA clusters. Other work in flies has identified a P-element that randomly transposes into different genomic regions rather than preferentially jumping into any pre-existing piRNA clusters [50, 51]. We identified several piRNA clusters that were conserved among species and, in particular, syntenic genomic loci encoding highly expressed piRNA clusters that are possibly attributable to specific chromatin structures or epigenetic information transmitted by maternal piRNAs [52]. These conserved piRNA clusters share no similarity between species, suggesting that hosts generated these highly diverse piRNA sequences in response to invasions by actively evolving TE families. The conserved synteny of specific piRNA clusters among species may reflect a mechanism for rapid activation of a piRNA-producing “factory” rather than a mode for preserving common sequences. However, the general expansion of piRNA clusters in which new clusters are gained more frequently than old clusters are lost has been reported in male germlines of different species [53], which aligns well with our findings in female germline cells. Therefore, although only a small portion of piRNA clusters is shared between oocytes and testis, our observations suggest that a similar mechanism of piRNA cluster turnover is likely present in both male and female gametes. The degeneration of piRNA clusters could be reasonably coupled with the loss of TE sequences in these regions. Once deleterious TEs have been suppressed, they could potentially go extinct, leaving only limited fragments behind in the genome, while the memory established in the piRNA clusters also would be gradually lost. Alternatively, these piRNA clusters could persist for considerably longer evolutionary periods to mitigate the potential mobilization of homologous TEs. However, some limitations should be considered when interpreting our results. In particular, not all oocytes collected from the 12 species reached the MII stage, when oocytes are most functional. Since the expression of small RNAs and TEs are likely stage-specific, more precise comparisons are warranted in future work using oocytes of the same stage between species.

## Conclusions

This large-scale oocyte small RNA profiling uncovers wide variation in small RNA composition in female germlines of different mammals, with piRNAs representing the predominant major small RNA population in most mammals. Our findings also reveal the widespread expression of PIWIL3 and its associated os-piRNAs among mammals. Finally, this study identifies conserved, syntenic, genomic locations containing numerous highly expressed, rapidly evolving piRNA clusters, suggesting a likely role in regulating TEs.

## Methods

### Animal use and care

Oocytes were collected from monkeys, mice, rats, golden hamsters, rabbits, guinea pigs, Chinese hamsters, dogs, zebrafish, pigs, and goats as described below. Mouse, rat, and zebrafish oocytes were obtained in compliance with the guidelines of the Center for Excellence in Molecular Cell Science at the Chinese Academy of Sciences (CAS). Monkey oocytes were collected following the guidelines of the Institute of Neuroscience, CAS. Chinese hamster oocytes were obtained in compliance with the guidelines of Shanxi Medical University.

### Monkey oocyte collection

Monkey oocytes were collected as previously described [16]. Superovulation was triggered in adult female *Macaca fascicularis* monkeys by intramuscular injection of 15 IU of GONAL-F recombinant human follitropin alfa (rhFSH) (Merck Serono, Germany) twice daily for 8 days and 1000 IU of human chorionic gonadotropin (hCG) (Sigma, USA) on the ninth day. Oocytes were collected by laparoscopic follicular aspiration 36 h after hCG injection. Cumulus cells were removed by adding hyaluronidase (2 mg/ml) and gently pipetting. Cumulus-free high-quality oocytes in metaphase II (MII) were visually inspected using a microscope, then cultured in HECM-9 medium at 37 °C with 5% CO_2_ for ~ 2 h. The MII oocytes were transferred into a droplet of acidic Tyrode’s solution (Sigma, T1788) to remove the zona pellucida, then washed twice with DPBS (Sigma, D5652). Each naked oocyte was transferred to a separate PCR tube containing < 0.5 μl DPBS and stored at − 80 °C until use.

### Mouse, rat, and golden hamster oocyte collection

Five-week-old female mice (C57BL/6), 6-week-old Sprague Dawley (SD) rats, and 5-week-old LVG strain golden hamsters were intraperitoneally injected with pregnant mare serum gonadotropin (PMSG) (Ningbo, China) (7.5 IU, 20 IU, and 20 IU, respectively). At 48 h after PMSG injection, mice were intraperitoneally injected with 7.5 IU hCG (Ningbo, China). At 56 h after PMSG injection, rats and golden hamsters were injected with 20 IU hCG. At 20 h after hCG injection, cumulus-oocyte complexes were collected from oviducts and placed in M2 medium (Sigma, M7167). Cumulus cells were removed by adding hyaluronidase (2 mg/ml; Sigma, H3506) and gently pipetting, and MII oocytes were selected and washed twice with DPBS. Each naked oocyte was transferred to a separate PCR tube containing < 0.5 μl DPBS and stored at − 80 °C prior to further use.

### Rabbit and guinea pig oocyte collection

Female rabbits weighing 2.5 kg (New Zealand) and 4-week-old female guinea pigs were provided by the Shanghai Songlian Lab Animal Farm. Rabbits and guinea pigs were administered 150 IU PMSG via intramuscular injection and 20 IU PMSG via intraperitoneal injection, respectively. At 56 h after PMSG injection, guinea pigs were intraperitoneally injected with 20 IU hCG; at 72 h after PMSG injection, rabbits were injected with 150 IU hCG via the ear vein. At 20 h later, animals were sacrificed, the ovaries were cut into pieces, and cumulus-oocyte complexes were released from the ovaries into M2 medium. Cumulus cells were removed by adding hyaluronidase (2 mg/ml) and gently pipetting. GV oocytes were transferred into acidic Tyrode’s solution to remove the zona pellucida, then washed twice with DPBS. Each naked oocyte was transferred to a separate PCR tube containing < 0.5 μl DPBS and stored at − 80 °C until use.

### Chinese hamster oocyte collection

Four-week-old female Chinese hamsters were sacrificed, then the ovaries were cut into pieces and cumulus-oocyte complexes were released from the ovaries into M2 medium. Cumulus cells were removed by adding hyaluronidase (2 mg/ml) and gently pipetting. GV oocytes were transferred into fresh M2 medium. Oocytes with normal form, bright color, and no cumulus cells were selected and each was transferred to a separate PCR tube containing < 0.5 μl DPBS, then stored at − 80 °C until use.

### Dog oocyte collection

MII oocytes were collected from 12- to 24-month-old beagles on the estimated ovulation day through continuous observation. The collected oocytes were transferred into M199 medium containing 25 mM HEPES. Cumulus cells were removed by adding hyaluronidase (2 mg/ml) with gentle pipetting. The oocytes were then washed twice in DPBS and each oocyte was transferred to a separate PCR tube containing < 0.5 μl DPBS and stored at − 80 °C until use.

### Zebrafish oocyte collection

Female AB strain zebrafish were anesthetized with 0.16 mg/ml Tricaine for 2–3 min, then transferred to phosphate-buffered saline (PBS) in a 35-mm plastic petri dish. Eggs were removed by gently pressing on the belly of each fish. Each egg was transferred to a separate 1.5-ml tube and stored at − 80 °C.

### Pig oocyte collection

Pig ovaries were obtained from a local slaughterhouse and transported to the laboratory in 0.9% NaCl (containing 100 IU/ml penicillin) within 2 h by Nanjing Zhushun Biotechnological Co., LTD. Antral follicles on the ovary surfaces were released by aspirating with an 8-gauge needle attached to a 10-ml syringe and washing three times in TCM 199 medium (Gibco, 11,150,059). Good quality cumulus-oocyte complexes with competent oocytes containing three cumulus cell layers were selected and cultured in TCM 199 medium supplemented with 0.1% polyvinyl alcohol (PVA), 3.05 mM D-glucose, 0.91 mM sodium pyruvate, 0.5 IU/mL follicle-stimulating hormone (FSH), 0.5 IU/mL luteinizing hormone (LH), 10 ng/mL epidermal growth factor (EGF), 75 μg/mL penicillin, 50 μg/mL streptomycin, 0.57 mM cysteine, and 10% follicular fluid. The cumulus-oocyte complexes were cultured in an incubator at 39 °C with 5% CO_2_ for 44 h. Cumulus-oocyte complexes were then transferred to M199 medium and cumulus cells were removed by adding hyaluronidase (2 mg/ml) and gently pipetting. MII oocytes with the first polar body were selected and transferred into acidic Tyrode’s solution to remove the zona pellucida, then washed twice with DPBS. Each naked oocyte was transferred to a separate PCR tube containing < 0.5 μl DPBS and stored at -80 °C until use.

### Goat oocyte collection

Goat ovaries were obtained from a local slaughterhouse and transported to the laboratory in 0.9% NaCl (containing 100 IU/ml penicillin) within 2 h by Nanjing Zhushun Biotechnological Co., LTD. Antral follicles on the surface of ovaries were released by aspirating with an 8-gauge needle attached to a 10-ml syringe and washed three times in TCM 199 medium (Gibco). Good quality cumulus-oocyte complexes with competent oocytes containing three cumulus cell layers were selected and cultured in TCM 199 medium supplemented with 10% fetal bovine serum (FBS, Gibco), 1 μg/ml 17 β-estradiol, 24.2 mg/L sodium pyruvate, 0.5 IU/ml FSH, 0.5 IU/ml LH, 10 ng/ml EGF, 100 mM cysteamine, and 200 mM cystine. The cumulus-oocyte complexes were cultured in an incubator at 38.5 °C with 5% CO_2_ for 24 h. Cumulus-oocyte complexes were then transferred into M199 medium and cumulus cells were removed by adding hyaluronidase (2 mg/ml) and gently pipetting. MII oocytes with the first polar body were selected and transferred into acidic Tyrode’s solution to remove the zona pellucida, then washed twice with DPBS. Each naked oocyte was transferred to a separate PCR tube containing < 0.5 μl DPBS and stored at − 80 °C until use.

### Human oocyte collection

Pre-matured oocytes in the germinal vesicle (GV) stage were donated by patients undergoing a cycle of intracytoplasmic sperm injection (ICSI). Oocytes were cultured in Continuous Single Culture medium (CSC, Irvine Scientific, CA, USA) with 10% Serum Substitute Supplement (SSS, Irvine Scientific) at 37 °C with 5% CO_2._ The presence of the germinal vesicle was confirmed in each oocyte under a macroscope 24 h later. The GV oocytes were then transferred into a droplet of acidic Tyrode’s solution (Sigma, T1788) to remove the zona pellucida, then washed twice with Dulbecco’s phosphate-buffered saline (DPBS) (Sigma, D5652). Each naked oocyte was transferred to a separate PCR tube containing < 0.5 μl DPBS and stored at − 80 °C prior to use.

### Western blot

Pooled MII oocyte samples (100 mouse oocytes, 75 rat oocytes, and 44 golden hamster oocytes) were each lysed in 8 μl of RIPA buffer (Beyotime, P0013C) with 1/100 proteinase inhibitor (Sigma, P8340) added. Samples were incubated on ice for 10 min, then 2 μl of 4 × protein loading buffer was added. Samples were incubated at 95 °C for 5 min, then resolved with 8% SDS-PAGE. Samples were then semi-dry transferred from the gel onto a polyvinylidene fluoride membrane and stained with anti-Dicer (Abcam, ab259327, 1:1000) or anti-tubulin (Proteintech, 66,031–1-Ig, 1:2000) antibodies overnight at 4 °C. Finally, membranes were incubated with appropriate horseradish peroxidase-conjugated secondary antibodies at room temperature for 1 h and developed using Eblot.

### NaIO4 oxidation of oocyte RNA

Total RNA was extracted from five MII or five GV oocytes from each species with TRIzol Reagent (Invitrogen, 15,596,026) after the addition of spike-in oligos. The RNA pellets were dissolved in 4 μl water, and 2 μl of each resulting sample was saved as a control. The remaining 2 μl of each RNA sample was treated with an oxidation reaction mixture, containing 1.5 μl 100 mM NaIO4 (Sigma, S1878), 2 μl of 5 × borate buffer (150 mM borax and 150 mM boric acid at pH 8.6), and 4.5 μl water. The reactions were incubated in the dark for 30 min at room temperature. RNA was then precipitated by adding 600 μl 100% ethanol with 25 μg linear acrylamide and 30 μl sodium acetate (3 M, pH 5.2) and incubating at − 30 °C for 30 min. Samples were centrifuged at 16,200 × *g* for 15 min at 4 °C. Pellets were washed twice with 75% ethanol and dissolved in 2 μl water for library construction.

### Generation of polyclonal antibodies for PIWIL3 and PIWIL1

Polyclonal antibodies used for immunoprecipitation were produced in rabbits by GL Biochem (Shanghai, China). Peptides with the following sequences were chosen as antigens to produce antibodies against PIWIL3 in guinea pig, rabbit, dog, pig, and goat, respectively: CQDLVVNTREKLRHVRHSKTG, C-EAQAVRALEAPQLHAREAE, CRHLEPQRHLEPQRHLEPQR, CAPEPAEPQPSEVARASEVT, and C-RTDPPLSFADLVRRGTAAQ. A peptide with the sequence C-HDLGVNTRQNLDHVKESK was used as the antigen to produce the anti-PIWIL1 antibody. Cysteine residues were included at the N terminal of the synthetic peptides for later coupling. The rabbits used for antibody production underwent seven rounds of immunization with the appropriate antigen prior to blood collection. Antibodies were then purified with peptide affinity chromatography.

### Immunoprecipitation of antibodies in transfected HEK293T cells

HEK293T cells were transiently transfected with plasmids encoding Flag-tagged PIWIL1, PIWIL2, or PIWIL3 using Lipofectamine 2000 (Invitrogen, 11,668,019) following standard procedures. Cells were harvested 36 h after transfection and incubated in lysis buffer (50 mM Tris–HCl [pH 7.4], 1% Polyoxyethylene (40) nonyl phenyl ether [NP-40], 150 mM NaCl, 0.5 mM Dithiothreitol [DTT], and 1 × proteinase inhibitor cocktail [Sigma]) for 10 min on ice. Cell lysates were centrifuged at 12,000 × *g* for 10 min at 4 °C. After removing 40 μl of supernatant to save as input, 400 μl of the supernatant of each sample was mixed with 3 μg of antibody specifically recognizing PIWIL3 coupled with Protein A agarose, then incubated overnight at 4 °C with gentle rotation. After washing the agarose with wash buffer (50 mM Tris–HCl [pH 7.4], 0.005% NP-40, and 150 mM NaCl) four times, immunoprecipitation (IP) pellets were boiled at 95 °C for 5 min with 1 × protein loading buffer. Anti-Flag-HRP (Sigma, A8592) was used to detect the target proteins via western blot.

### RNA immunoprecipitation of PIWI proteins in oocytes

Protein A Magnetic Beads (NEB, S1425S) were washed twice with RIP lysis buffer (50 mM Tris–HCl [pH 7.4], 150 mM NaCl, 1 mM EDTA, 0.5% NP-40, 0.5 mM DTT, and 0.1 U/μl RNase Inhibitor) containing 0.4 U/μl proteinase inhibitor cocktail (Sigma). For immunoprecipitation of PIWI protein for each sample, the washed beads derived from 2.5 μl of the original bead slurry were mixed with 1.5 μg antibody to a total volume of 10 μl and incubated on a rotator at room temperature for 0.5 h. Coupled beads were washed with RIP buffer before use. Oocyte samples were pooled for extraction for some species, namely guinea pig (six GV oocytes), goat (10 MII oocytes), pig (10 MII oocytes), and rat (nine MII oocytes). The pooled samples were lysed by adding 30 μl, 30 μl, 40 μl, or 22.5 μl cold RIP lysis buffer, respectively, in a DNA low-bind tube (Eppendorf, Germany) and incubating on ice for 10 min. Samples were then centrifuged at 16,200 × *g* for 5 min at 4 °C. Each 7.5 μl sample of oocyte lysate was transferred to a PCR tube that contained antibody-coupled beads for RIP assays. RNA was extracted with TRIzol from the remaining oocyte lysate in each tube after distribution for IP reactions. For IP reactions, after rotating for 5 h at 4 °C, beads were washed with RIP lysis buffer by rotating at room temperature for 5 min; this was repeated three times and followed by another wash with PBS. The beads were resuspended in 2 μl water and incubated at 75 °C for 5 min. After cooling on ice, the tubes were placed on a magnetic stand (Thermo Scientific, 12321D) for 10 s, and the supernatants were recovered for RNA extraction and sequencing library construction.

### Small RNA library construction

Small RNA libraries were constructed from single oocytes as previously described [33]. Briefly, each single oocyte was lysed and incubated at 72 °C for 3 min, then cooled on ice. After 3′ adapter ligation, samples were incubated with 5 U of lambda exonuclease and 25 U of 5′ de-adenylates. Small RNAs were reverse-transcribed after 5′ adapter ligation. Two rounds of amplification were conducted to obtain the libraries, which were recovered on a 6% polyacrylamide gel.

### mRNA library construction

mRNA library construction was conducted as previously described [33]. Briefly, a single oocyte was lysed and incubated at 72 °C for 3 min, then cooled on ice. Next, 1 μl of a 1/500,000–1/50,000 dilution of the ERCC RNA Spike-In Mix (Invitrogen, 4,456,740) was added to each sample. After reverse transcription and PCR pre-amplification, cDNA was purified and 3 ng used for a tagmentation reaction with Tn5 transposase. Amplified libraries were recovered and sequenced as described above.

### Phylogenetic species tree

A phylogenetic tree was built for the 12 representative vertebrate species with TimeTree [54] and visualized with ggtree [55].

### Sources of RNA sequences and genome assemblies

miRNA sequence annotations were downloaded from miRbase (Version 22) (http://www.mirbase.org/). mRNA, lncRNA, rRNA, snoRNA, and tRNA sequences were downloaded from Ensembl Genes (www.ensembl.org, version 100) and the Genomic tRNA Database (http://lowelab.ucsc.edu/GtRNAdb). The reference genome sequences, repeat annotations (RepeatMask files), and genomic annotations (Gene Transfer Format) were downloaded from the US National Center for Biotechnology Information (NCBI) RefSeq database. The accession or version numbers were as follows: GRCh38.p13 (human), Macaca_fascicularis_5.0 (monkey), OryCun2.0 (rabbit), Cavpor3.0 (guinea pig), GRCm38.p6 (mouse), Rnor_6.0 (rat), MesAur1.0 (golden hamster), CriGri-PICRH-1.0 (Chinese hamster), CanFam3.1 (dog), ARS1 (goat), Sscrofa11.1 (pig), and GRCz11 (zebrafish). The consensus TE sequences were downloaded from Repbase [56]. RepeatModeler2 was also used to predict and classify consensus TEs using the default parameters [57].

### Oocyte small RNA-Seq

High-throughput RNA sequencing was performed on a NovaSeq 6000 (PE150). Because small RNAs typically range from 18–40 nt in length, we only used the R1 FASTQ files for subsequent analyses. Cutadapt was used to clip adaptor sequences and to filter out low-quality reads [58]. Reads that failed to match the adaptor sequences or that were shorter than 17 nt in length were discarded. Redundant sequences were collapsed as high quality reads (Gordon A, Hannon GJ: Fastx-toolkit. FastQ/A short-reads pre-processing tools, unpublished) and mapped to the reference sequences using bowtie [59]. The resulting reads were classified as known miRNAs, tRNA-derived small non-coding RNAs (tsRNAs), rRNA-derived small non-coding RNAs (rsRNAs), small snoRNAs, lncRNAs, and mRNAs, successively. Reads that could not be mapped to these known small RNAs were used in successive piRNA and endo-siRNA prediction. Sequences that were not annotated as any of the RNA categories described above were classified as “others.”

### Oocyte mRNA-Seq

Trimmomatic [60] was used to clip adaptor sequences and to filter out low-quality reads. The high-quality reads were mapped to reference genomes using STAR [61]. Gene expression levels were calculated as transcripts per kilobase of exon model per million mapped reads (TPM) with StringTie [62].

### piRNA sequence logos

The sequence logos for nucleotide positions 1–10 in piRNAs were calculated with weblogo [63].

### Ping-pong signatures

The score for a distance of *x* nt was calculated as follows:

$$\sum ({M}_{i}, {N}_{i}+x)$$

where M_i_ is the number of reads with piRNA 5′ ends located at position* i* and *N*_*i*+*x*_ is the number of reads with piRNA 5′ ends located at position *i* + *x* (opposite strand). When *x* = 0, the 5′ ends of two piRNAs had no overlap. When *x* = 10, the 5′ ends of two piRNAs had a 10-nt overlap (ping-pong). For analyses including multi-mappers, reads were apportioned based on the number of times they could be aligned to the genome. In ping-pong analyses, overlaps at nt positions 1–9 and 11–20 were used as the background to calculate Z-scores.

### Prediction of piRNAs and piRNA clusters

Small RNAs that could not be mapped to known annotations were used for piRNA cluster prediction using previously described methods [64]. To remove potential noise, we used stringent parameters in proTRAC (v2.4.2) to identify piRNA clusters in these 12 species (-1Tor10A 0.75 -1Tand10A 0.5). Due to differences in the length distribution of small RNA sequences in these species, we set the piRNA length limit to 17–23 nt (-pimin 17 -pimax 23) in rabbits and guinea pigs. In mice, rats, Chinese hamsters, and zebrafish, we set the piRNA length limit to 24–32 nt (-pimin 24 -pimax 32). In humans, monkeys, golden hamsters, dogs, goats, and pigs, we set the piRNA length limit to 17–32 nt (-pimin 17 -pimax 32). Other parameters in proTRAC were not changed, and the default parameters were used. Unknown reads of 17–32 nt in length located in these clusters were defined as piRNA candidates.

### Prediction of endo-siRNAs and endo-siRNA clusters

Endo-siRNA clusters were defined as previously described [16]. Briefly, we used proTRAC (v2.4.2) to identify endo-siRNA clusters in mouse oocytes (-pimin 17 -pimax 24 -1Tor10A 0.25 -1Tand10A 0.1 -pdens 0.01 -clstrand 0.5). The resulting coordinates of endo-siRNA clusters are listed in Additional file 2: Table S18. Compared to piRNAs, endo-siRNAs have no preference for 10A, and the preference for 1U is less significant. Therefore, reads that could not be mapped to known small RNAs (miRNAs, tsRNAs, rsRNAs, and sn/snoRNAs) and were 17–24 nt in length were used to predict candidate endo-siRNA clusters, which were classified as those that met two criteria: (1) more than 75% of the reads were 21–23 nt in length and (2) at least four sequences in the cluster had a 2-nt overhang and the percentage of sequences having a 2-nt overhang in the cluster was > 10%. Reads 17–24 nt in length that could be mapped to these endo-siRNA clusters were predicted to be endo-siRNAs.

### miRNA families

We compared miRNA expression levels in the 12 representative vertebrate species using miRNA families. miRNA family annotations were downloaded from TargetScan (Release 7.2) [65]. When small RNAs were mapped to the miRNA families, there were 0 mismatches allowed. Expression levels of miRNA families were qualified by size factor [66].

### Synteny analysis

Synteny analysis was performed using liftOver in the UCSC toolkit (http://genome.ucsc.edu/cgi-bin/hgLiftOver). Human (hg38) piRNA clusters were aligned to monkey (macFas5), rabbit (oryCun2), guinea pig (cavPor3), mouse (mm10), rat (rn6), golden hamster (mesAur1), Chinese hamster (criGriChoV2), dog (canFam3), pig (susScr11), goat (oviAri4), and zebrafish (danRer11) genomes using the command “liftOverminMatch = 0.1”. piRNA-producing clusters in the human genome that had been successfully identified in another genome were considered syntenic. Loci producing piRNAs from a syntenic location in another species were considered to be evolutionarily conserved.

### PhyloP scores calculation

ClustalW2 was used for multiple sequences alignment [67]. We used the likelihood ratio test (LRT) method and CONACC mode from phyloP to compute conservation scores for each site in the alignment [68]. We then divided the site equally into 100 bins according to their coordinates and calculated average phyloP scores for each bin. The values represent -log *p*-values under a null hypothesis of neutral evolution. The sites predicted to be conserved are assigned positive scores while sites predicted to undergo accelerated evolution are assigned negative scores.

### Heatmap and principal component analyses

A heatmap was plotted using the “pheatmap” package in R [69]. PCA was performed using the *prcomp* function in R and visualized using the “ggplot2” package [70].

### Small-RNA spike-ins and normalization

Three unmethylated and three methylated small-RNA spike-ins were synthesized by Integrated DNA Technologies (IDT) and pooled. The sequence of each spike-in can be found in our previous study [16]. To quantify the expression levels of small RNAs in the RNA IP assays and NaIO4 oxidation data, we normalized the read counts of small RNAs to those of the spike-ins.

### Mapping piRNA and RNA-Seq data to TEs

To measure the expression levels of TE-related piRNAs, piRNAs were mapped to consensus TEs. For analyses including multi-mappers, reads were classified based on the number of times they could be aligned to the TEs. We quantified the TE-related piRNA expression levels in reads of exon model per million mapped fragments (RPM). Oocyte RNA-Seq data were mapped to the consensus TEs using bowtie2 [71]. TE mRNA expression levels were quantified in fragments per kilobase of exon model per million mapped fragments (FPKM).

### TE genomic features

BED files containing repetitive elements predicted by RepeatMasker were downloaded from NCBI RefSeq. The genomic features of average TE divergence and TE copy numbers were calculated based on the BED files. Pearson’s correlation coefficients for piRNA, TE mRNA abundance, and genomic features were calculated using the “corrplot” package in R [72].

### Supplementary Information

**Additional file 1.** Supplementary figures. Figure S1–S12.

**Additional file 2.** Supplementary tables. Table S1–S18.

**Additional file 3.** Uncropped images for the blots in Fig. 1, Additional file 1: Fig S4–S5.

**Additional file 4. **

## Data Availability

The deep-sequencing data have been deposited at the NCBI Gene Expression Omnibus (GEO) (http://www.ncbi.nlm.nih.gov/geo/) database under accession number GSE200470 and GSE256514 [73, 76]. The original code and processed data have been uploaded and is available on GitHub repository (https://github.com/liuwell/oocytes_smallRNA) [74]. The version of the code used in this study was archived in the Zenodo repository under the accession code https://doi.org/10.5281/zenodo.10682583 [75].
