# Supplementary material for: Divergent composition and transposon-silencing activity of small RNAs in mammalian oocytes
Source: Genome Biol. 2024 Mar 26;25:80. doi: 10.1186/s13059-024-03214-w (PMC10964541; doi:10.1186/s13059-024-03214-w)
Supplement: Supplementary file 1 — Additional file 1. Supplementary figures. Figure S1–S12. [file 13059_2024_3214_MOESM1_ESM.docx]

**Additional file 1: Supplementary figures**


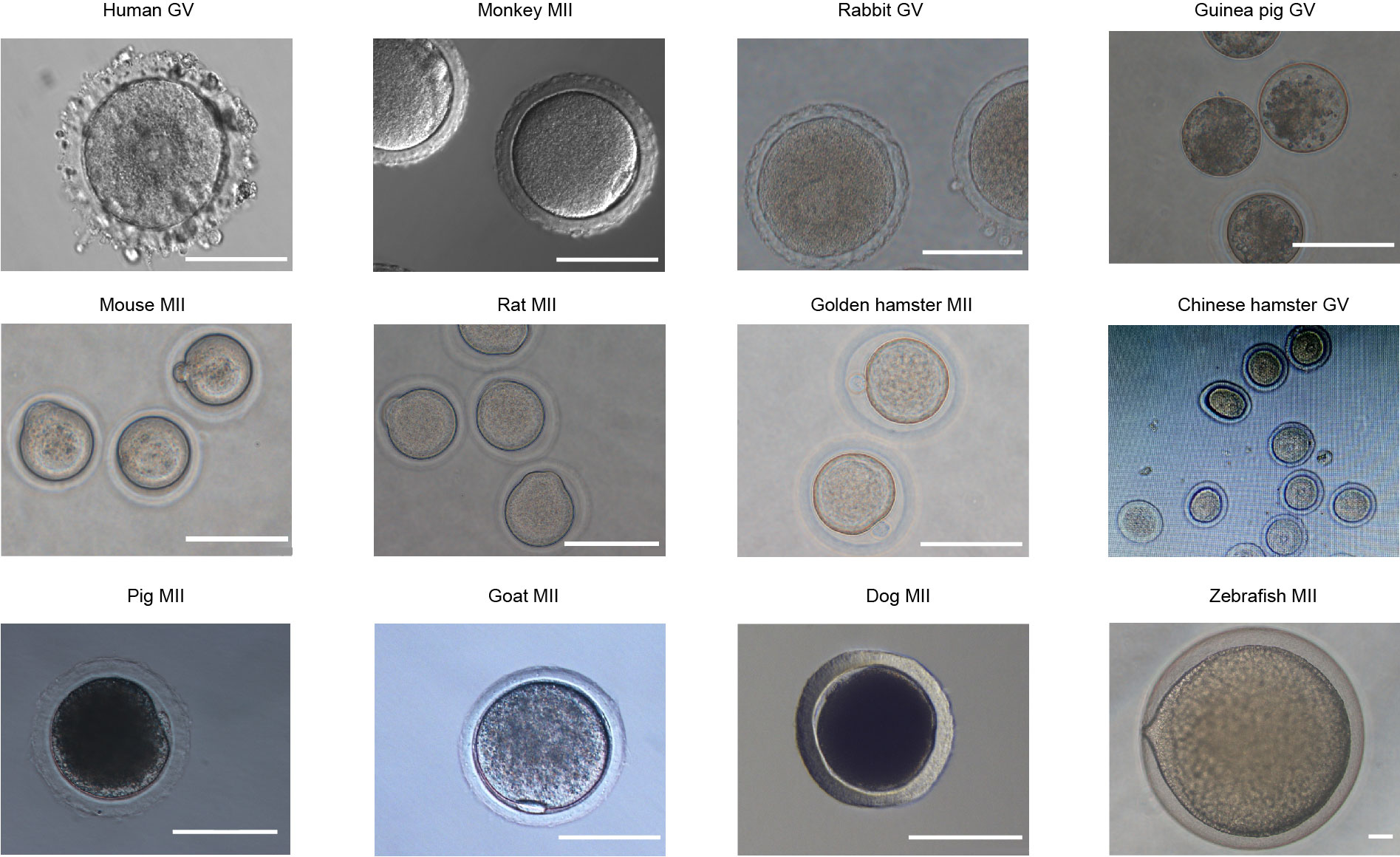


**Fig. S1: Images of oocytes collected from 12 representative vertebrate species.**

Microscope images of oocytes collected from 12 species. The developmental stage of each oocyte is noted above the figure. MII, metaphase II stage; GV, germinal vesicle stage. Scale bar = 100 μm.


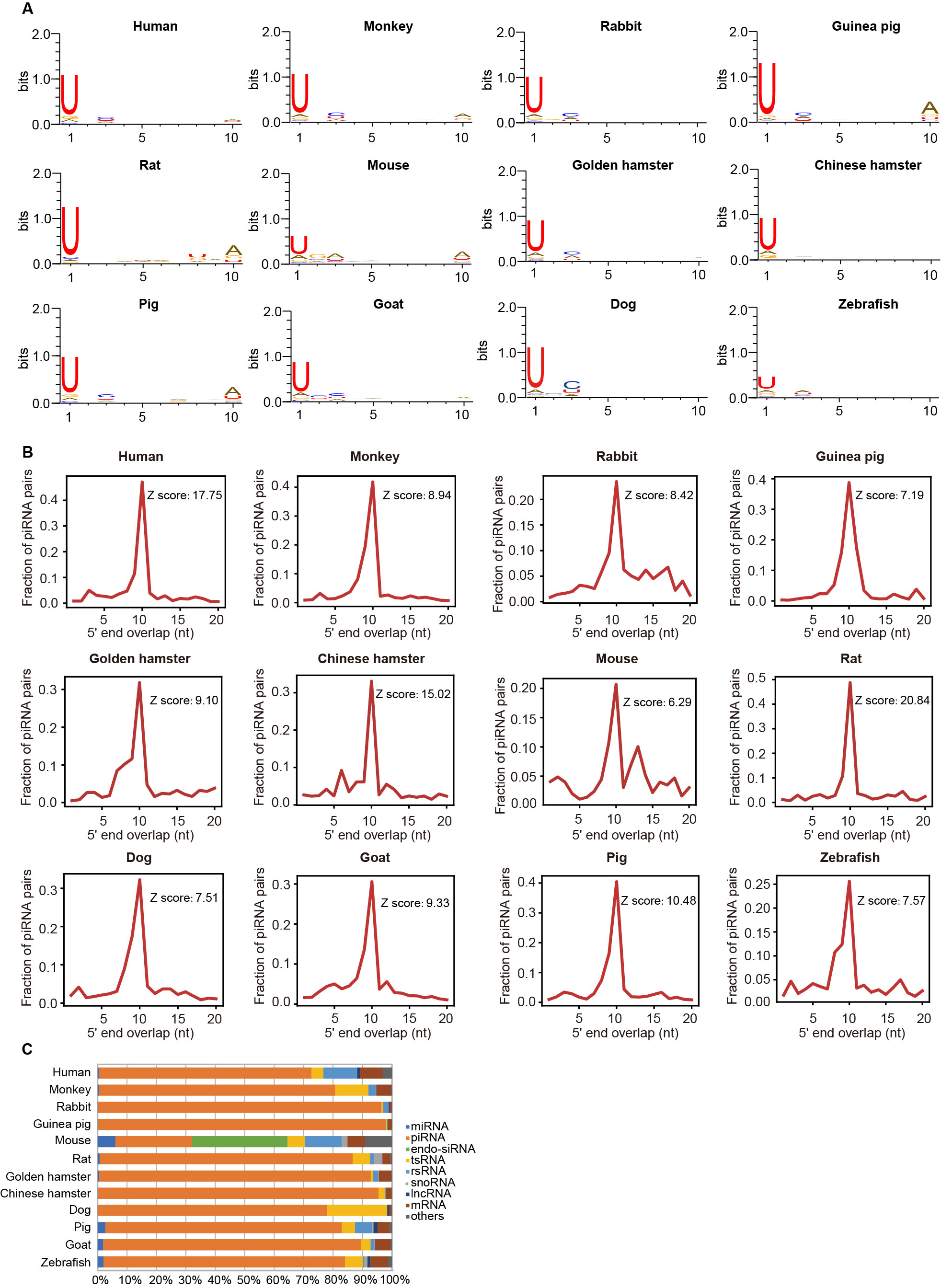


**Fig. S2: Basic analysis of oocyte piRNAs.**

**A** Nucleotide bias in positions 1-10 of oocyte piRNAs. **B** Ping-pong signatures of oocyte piRNAs. The Z-scores corresponding to ping-pong signatures are shown to the right of each peak. **C** Small RNA composition in oocytes from each of the 12 vertebrate species.


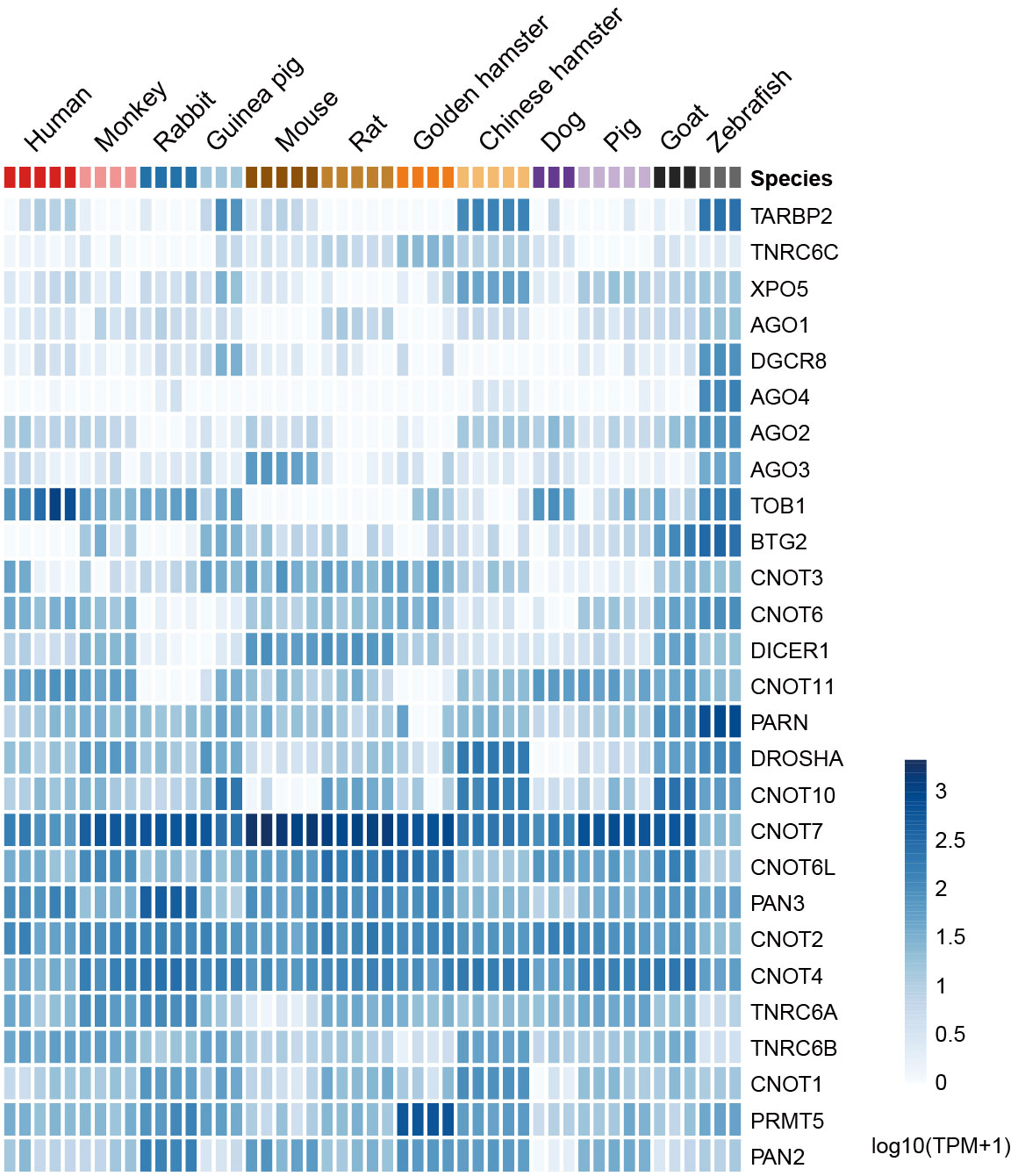


**Fig. S3: Expression of miRNA/endo-siRNA pathway genes in the 12 species.**

Expression levels of miRNA/endo-siRNA pathway genes detected in RNA-Seq data of oocyte samples from 12 species. Hierarchical clustering was used to generate a heatmap (method=‘complete’).


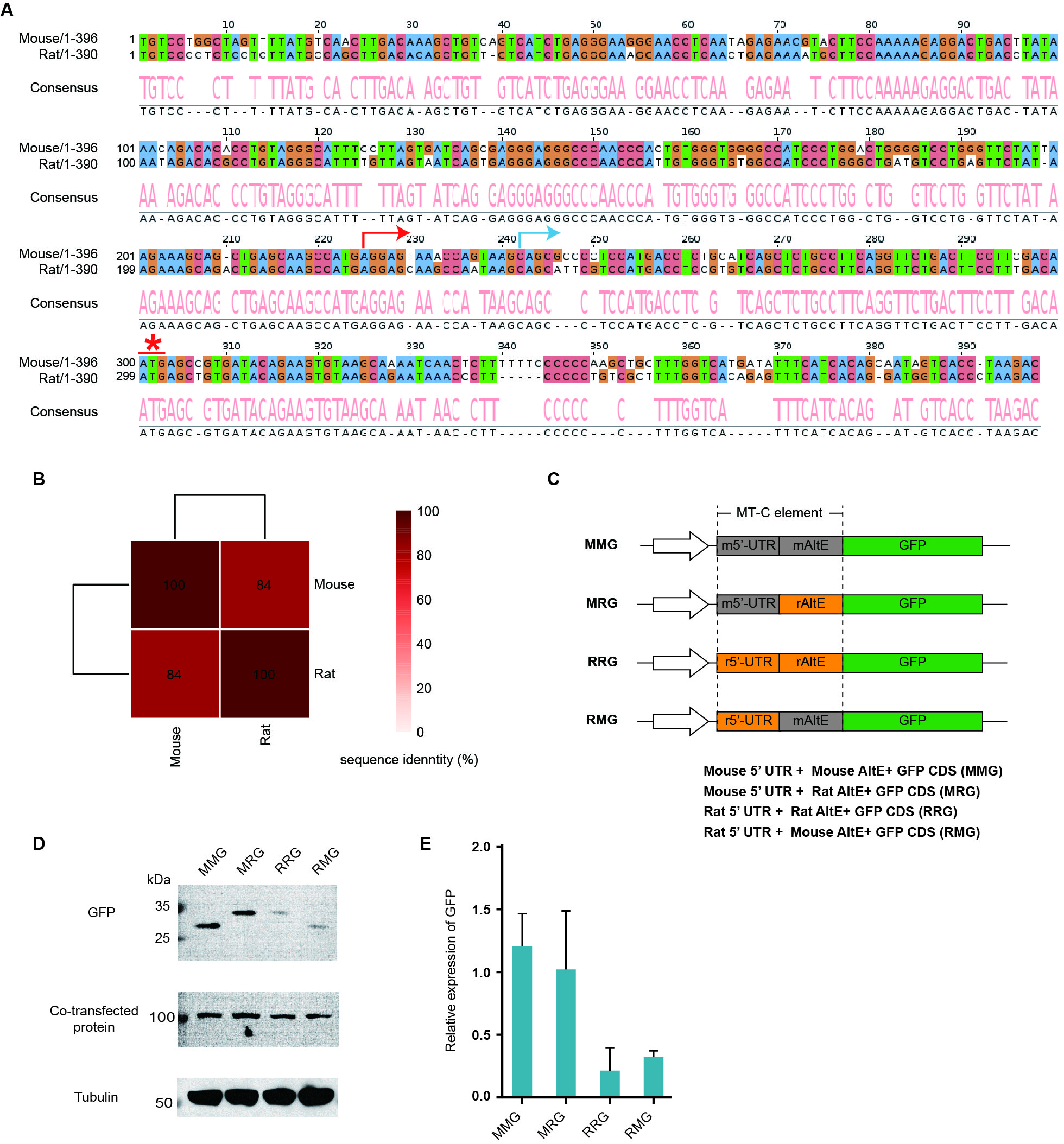


**Fig. S4: The insertion of MT-C elements in the 5’ UTR of rat DicerO might reduce its expression.**

**A** Multiple sequence alignment of the MT-C element from mouse and rat. Red (mouse), blue (rat), arrows indicate the TSS of DicerO transcripts. **B** Heatmap of sequence identity for the MT-C element between mouse and rat. The percent of sequence identity is shown for each comparison. **C** A GFP reporter fused with different combinations of the MTC-containing 5’UTR and AltE of DicerO transcripts from mice or rats. CMV promoter is used to drive transgene expression in 293T cells. **D** Western blot showing the expression of GFP protein fused with different combinations of 5’UTR and AltE from mouse or rat. The assays were independently repeated three times with similar results and a representative result is shown. **E** Quantification of Western blots for GFP expression, normalized to Flag-PIWIL2 co-transfection, across three replicates.


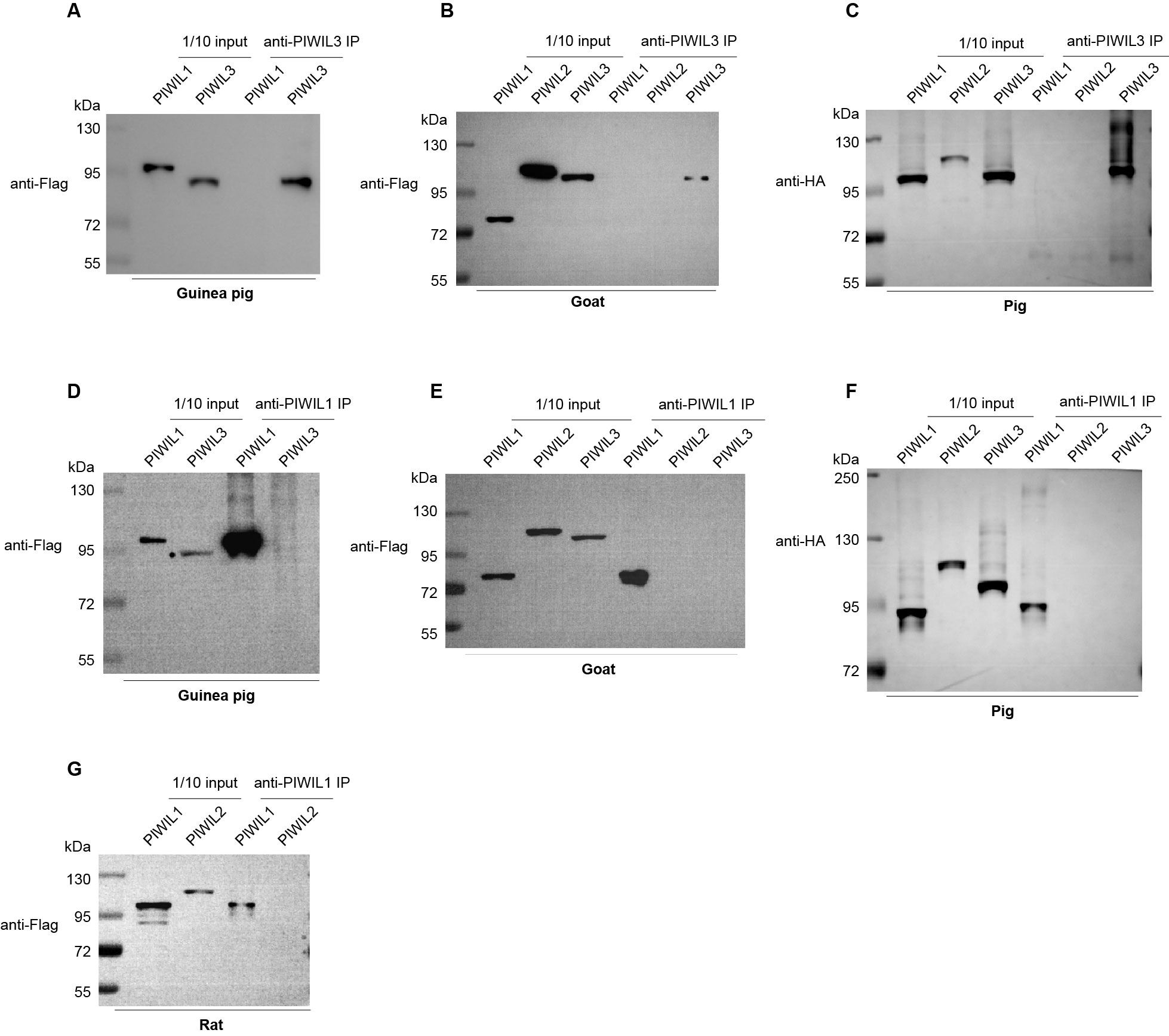


**Fig. S5: PIWIL1 and PIWIL3 immunoprecipitation.**

**A**-**C** Examination of the specificity and efficiency of antibodies for immunoprecipitation of PIIWL3 protein from guinea pig (**A**), goat (**B**), and pig (**C**) overexpressed in transfected HEK293T cells. **D-G** Examination of specificity and efficiency of antibodies for immunoprecipitation of PIIWL1 protein from guinea pig (**D**), goat (**E**), pig (**F**), and rat (**G**) overexpressed in transfected HEK293T cells.


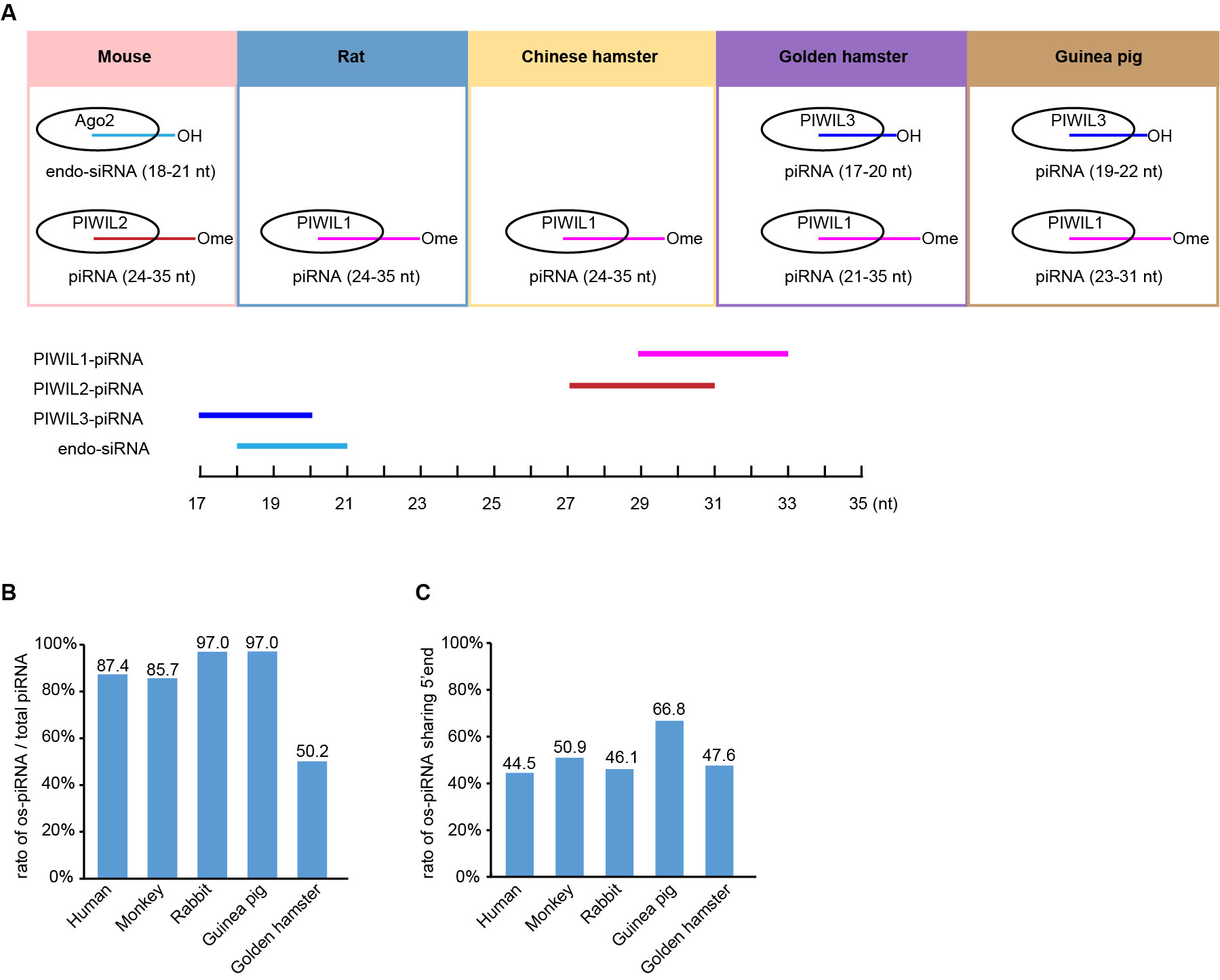


**Fig. S6:** **The relationship between os-piRNAs and long piRNAs.**

**A** Comparison of piRNA and endo-siRNA expression in the five rodent species included in this study: mouse, rat, Chinese hamster, golden hamster, and guinea pig. **B** Percentage of os-piRNAs (by count) out of total piRNAs in five species: human, monkey, rabbit, guinea pig, and golden hamster. **C** Percentage of os-piRNAs (by count) sharing the same 5’ ends with long piRNAs in five species: human, monkey, rabbit, guinea pig, and golden hamster.


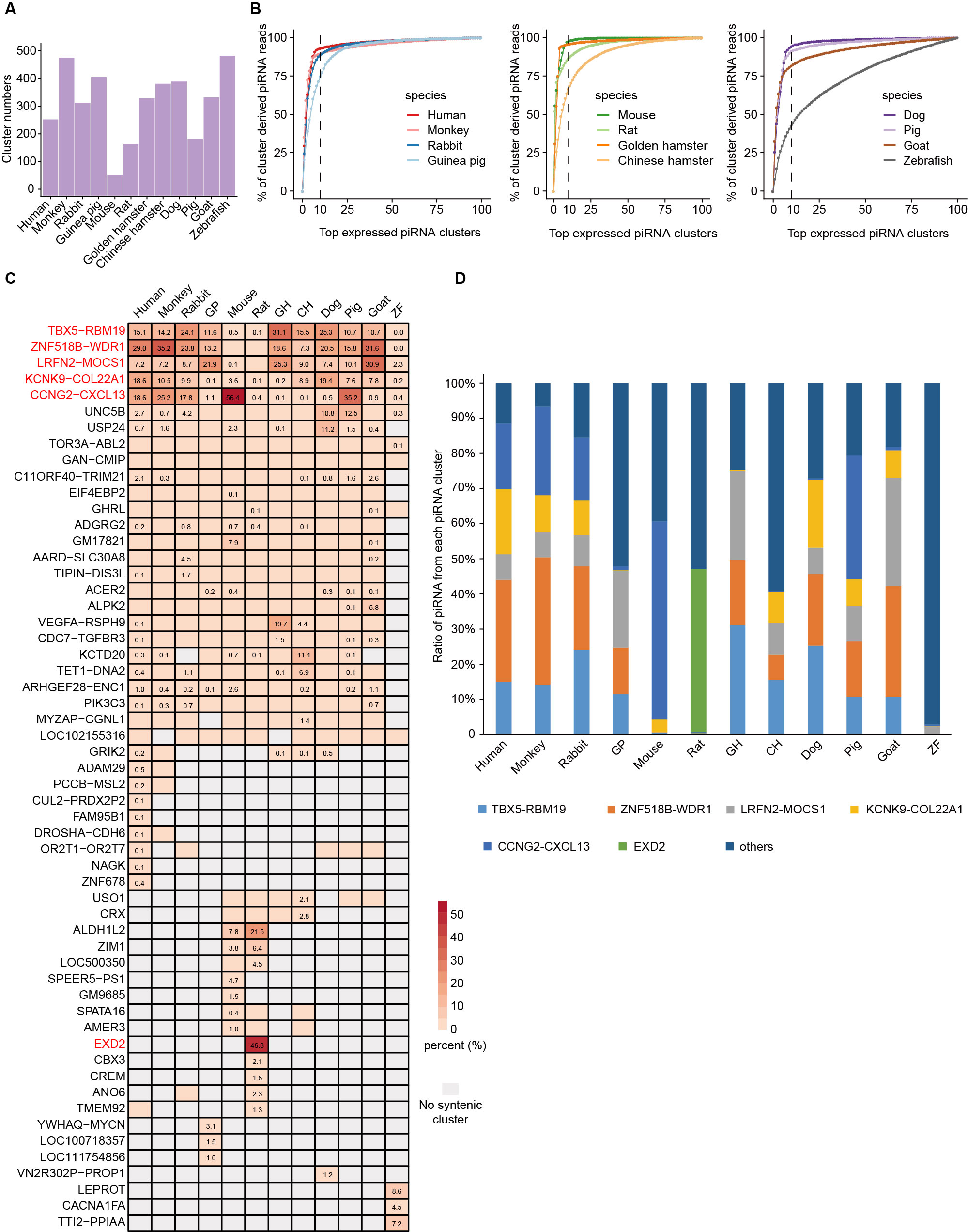


**Fig. S7:** **Analysis of homologous piRNA clusters.**

**A** Number of piRNA clusters identified in each species. **B** Cumulative distribution of piRNA reads derived from the 100 most highly expressed piRNA clusters in each species. **C** Ratio of piRNA reads derived from the 56 oocyte piRNA clusters existing in at least one species. The five most highly expressed syntenic piRNA clusters and the most highly expressed piRNA cluster in rat are shown in red text. **D** The contributions of the five most highly expressed syntenic piRNA clusters and the most highly expressed piRNA cluster in rat (piC-EXD2) are shown for each species.


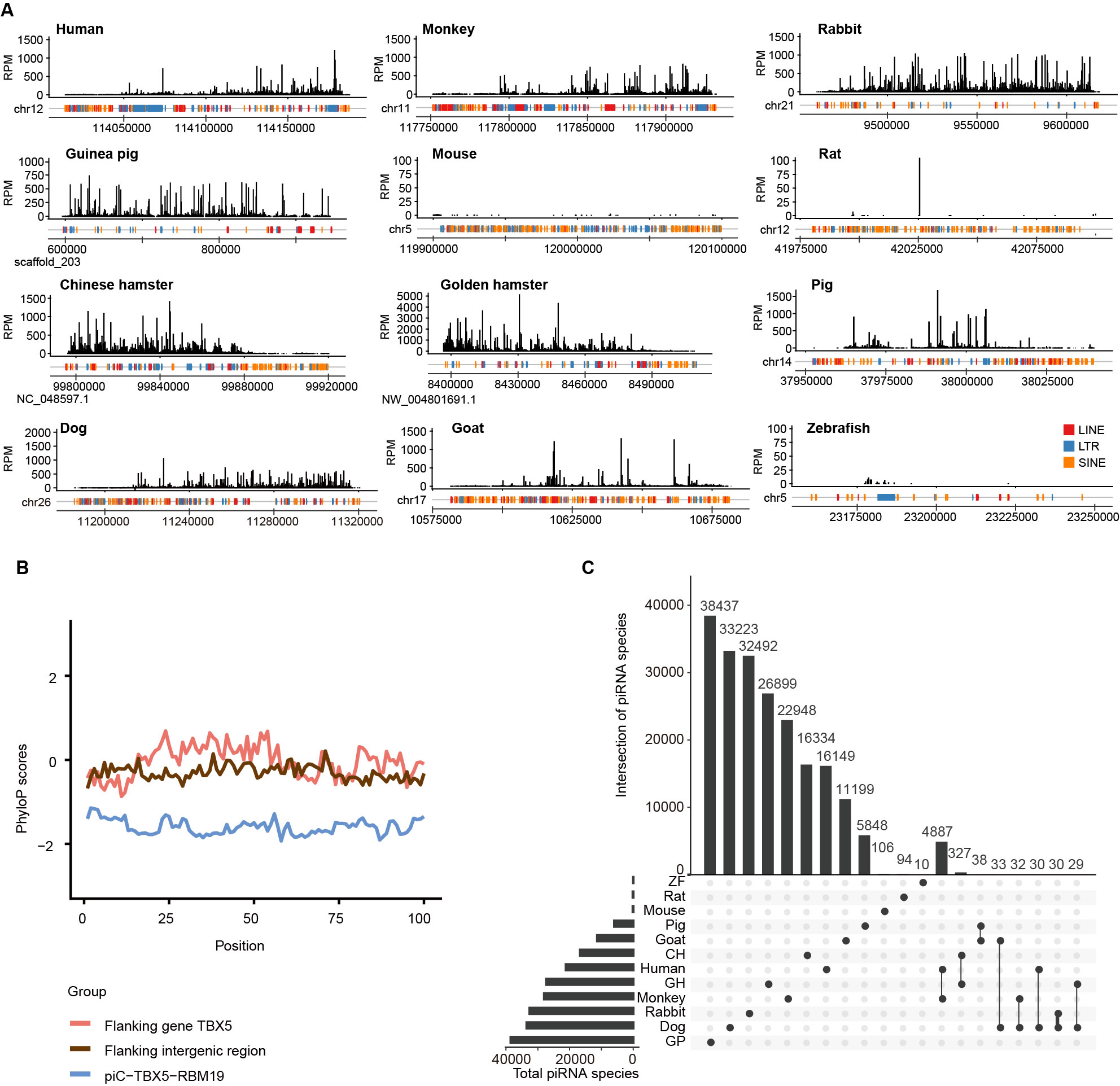


**Fig. S8:** **Analysis of homologous piRNA clusters.**

**A** Genome browser snapshots of piC-RBM19-TBX5 in the 12 representative vertebrate species. The black peaks indicate piRNA abundance (quantified in RPM). **B** The phyloP scores of piRNA cluster piC-RBM19-TBX5, its flanking intergenic region, and cDNA sequences of its flanking protein-coding genes. The values represent -log p-values under a null hypothesis of neutral evolution. The sites predicted to be conserved are assigned positive scores while sites predicted to undergo accelerated evolution are assigned negative scores. **C** UpSet plot showing the identities of piRNA sequences in the piC-RBM19-TBX5 cluster among the 12 vertebrate species. The intersections of piRNA species are indicated by lines linking samples in the lower panel. piRNAs were classified as the same in multiple species if the piRNA length was < 23 nt and had ≤ 1 mismatch, or if the length was > 23 nt and had ≤ 2 mismatches. No mismatches were allowed in the seed region (2-8 nt). GP, guinea pig; GH, golden hamster; CH, Chinese hamster; ZF, zebrafish.


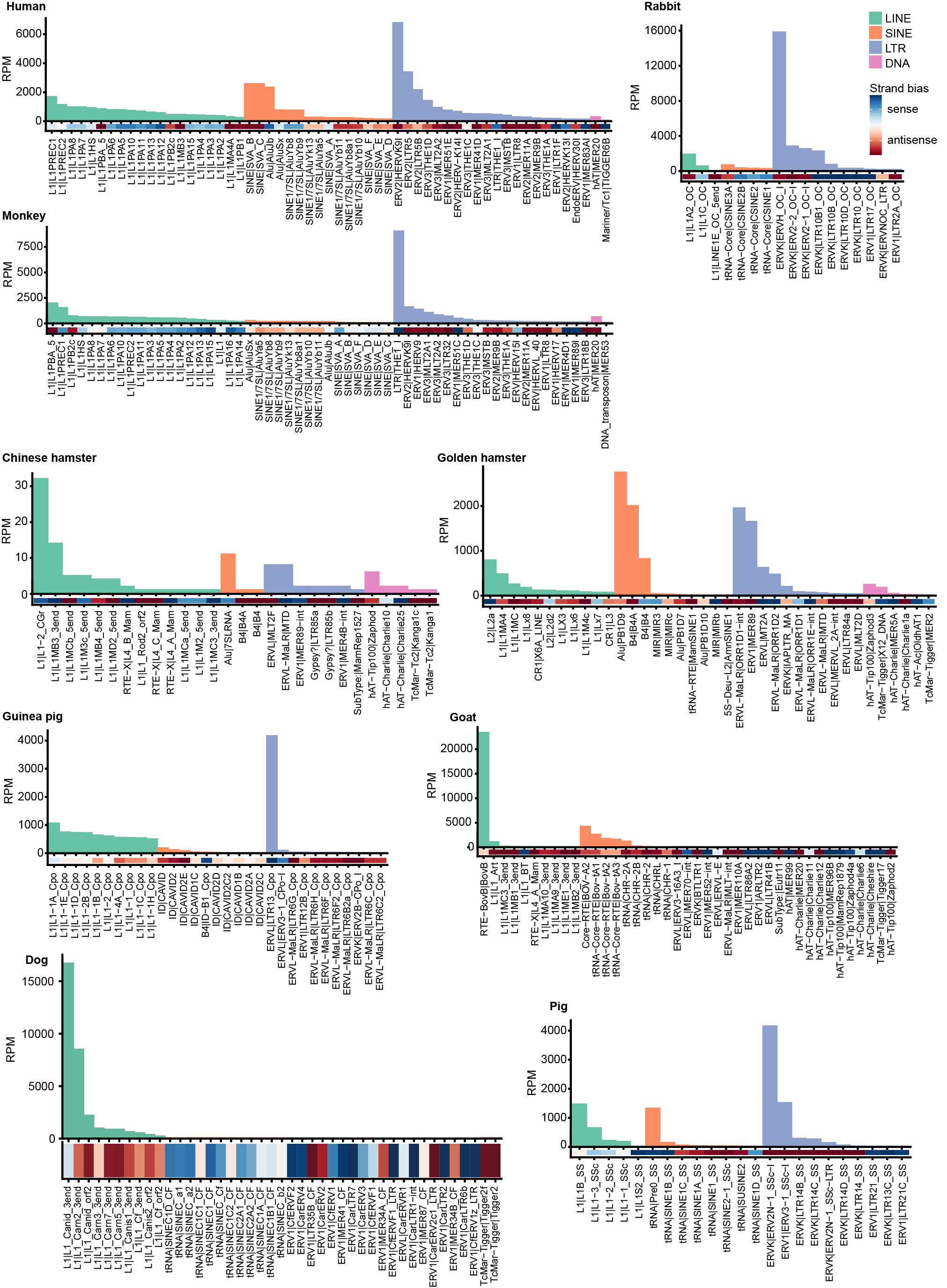


**Fig. S9:** **TE analysis in the syntenic oocyte piRNA cluster piC-ZNF518B-WDR1.**

Expression levels of piRNAs (quantified in RPM) mapping to different TE subfamilies (LINEs [green], SINEs [orange], LTRs [blue], and DNA transposons [pink]) in the piC-ZNF518B-WDR1 cluster in nine species: human, monkey, rabbit, guinea pig, golden hamster, Chinese hamster, dog, goat, and pig.


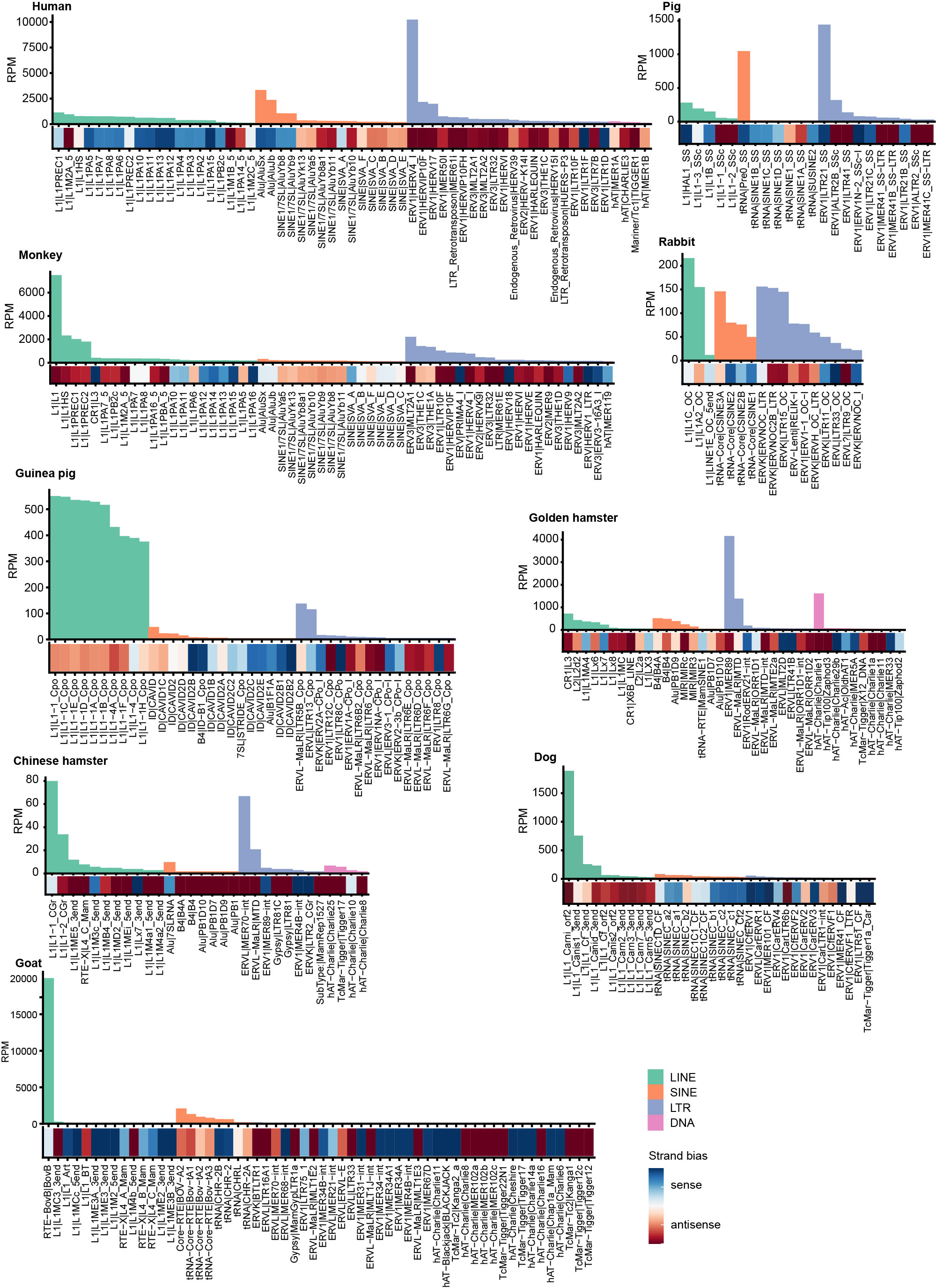


**Fig. S10:** **TE analysis in the syntenic oocyte piRNA cluster piC-RBM19-TBX5.**

Expression of piRNAs (quantified in RPM) mapping to different TE subfamilies (LINEs [green], SINEs [orange], LTRs [blue], and DNA transposons [pink]) in the piC-RBM19-TBX5 cluster in nine species: human, monkey, rabbit, guinea pig, golden hamster, Chinese hamster, dog, goat, and pig.


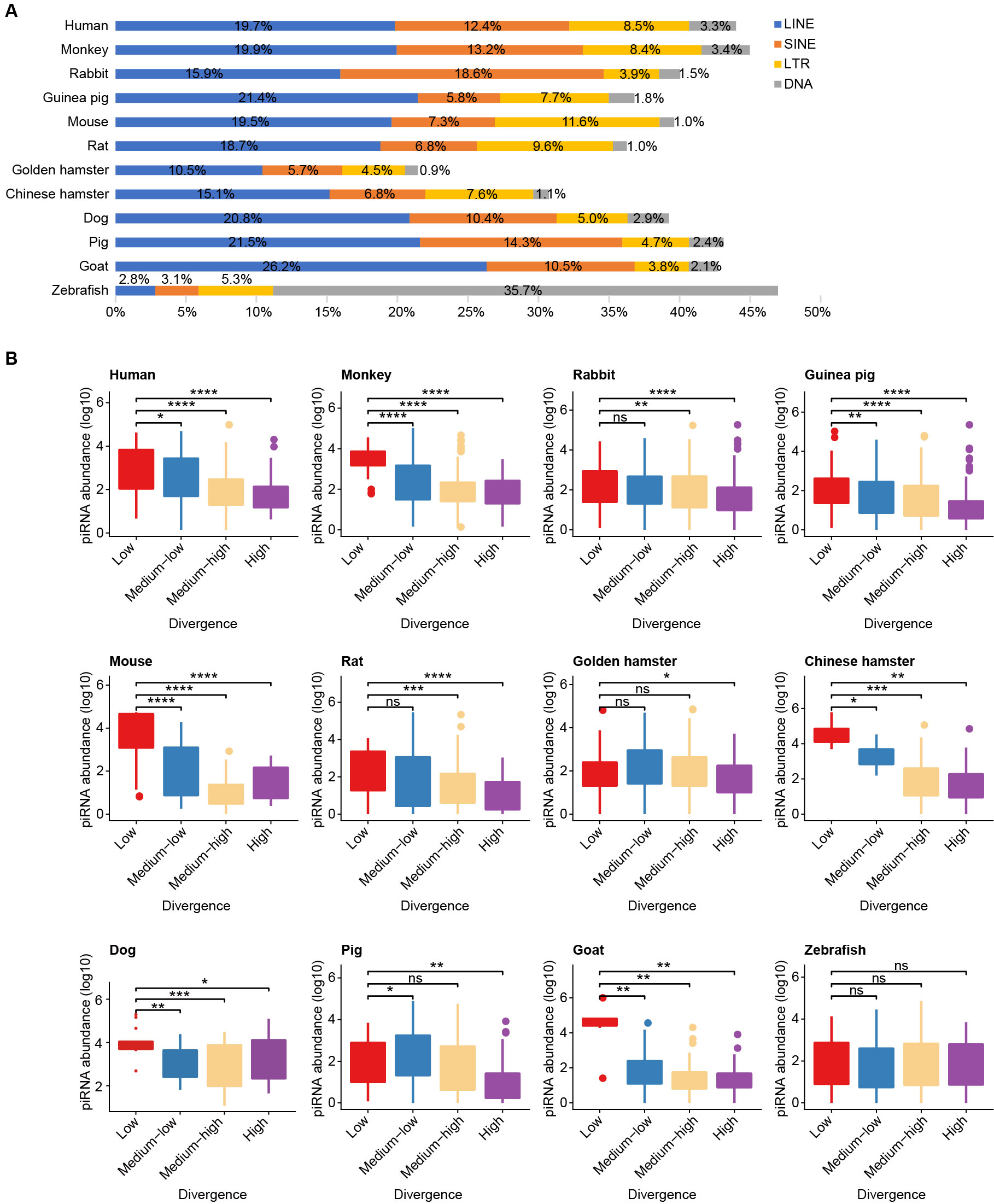


**Fig. S11:** **Correlations between piRNA abundance and TE divergence rate.**

**A** Proportions of different types of TEs in the genomes of the 12 representative vertebrate species. **B** Box plot showing the abundance of TE-related piRNAs and the corresponding TE divergence. TEs were equally divided into four groups based on TE divergence. **p* ≤ 0.05, ***p* ≤ 0.01, ****p* ≤ 0.001, *****p* ≤ 0.0001 (Student’s *t*-test). ns, not significant.


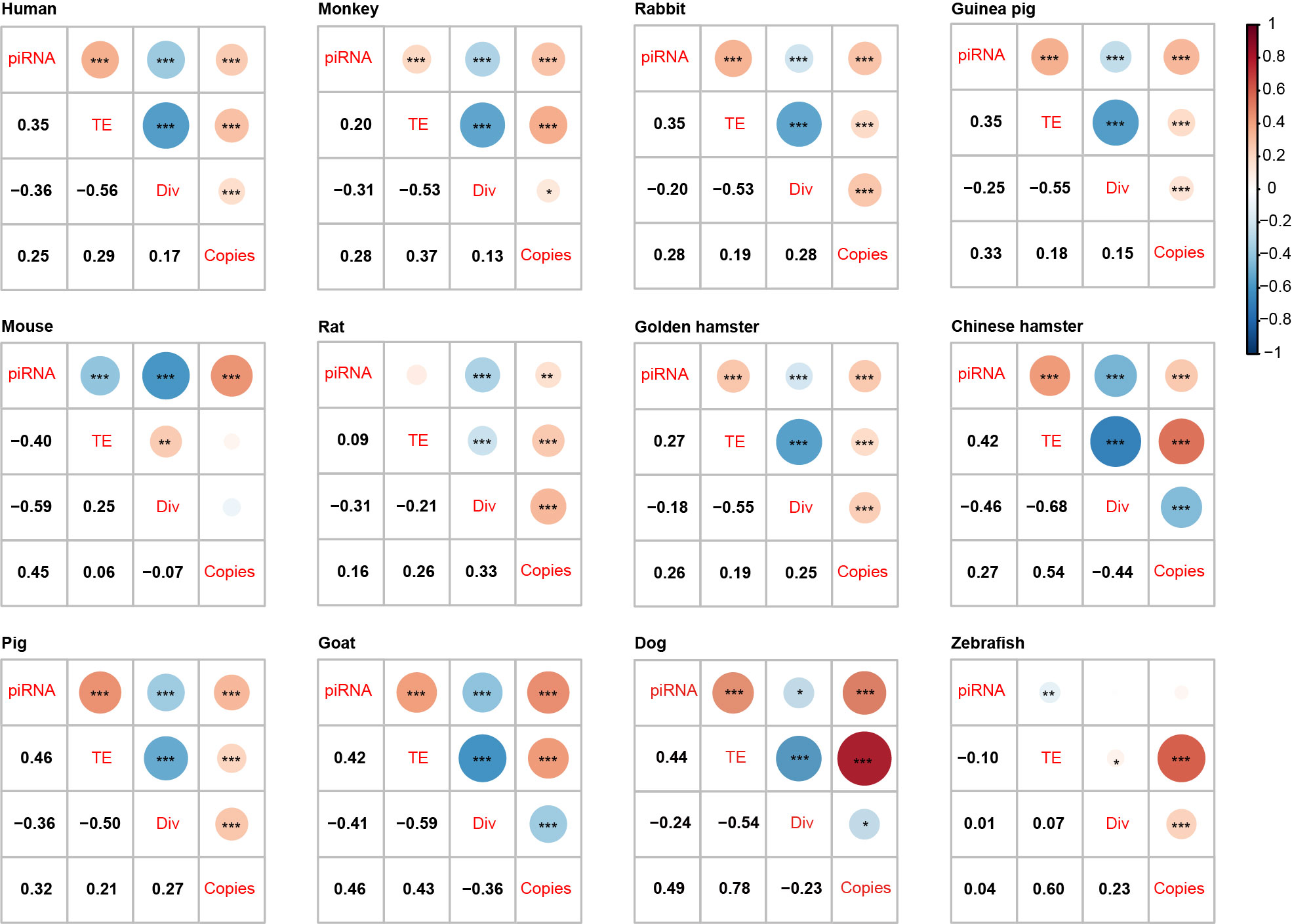


**Fig. S12:** **Correlations between piRNA abundance and TE features.**

Pearson’s correlation coefficient for piRNA abundance, TE mRNA levels, and TE genomic features (divergence rate and copy number). Red and blue indicate positive and negative correlations, respectively. **p* < 0.05, ***p* < 0.01, ****p* < 0.001 (pairwise *t*-test). piRNA, TE-related piRNA abundance; TE, TE mRNA expression levels; Div, average divergence of TE families; Copies, total copy number of TE families in the genome.
