# Supplementary material for: Divergent composition and transposon-silencing activity of small RNAs in mammalian oocytes
Source: Genome Biol. 2024 Mar 26;25:80. doi: 10.1186/s13059-024-03214-w (PMC10964541; doi:10.1186/s13059-024-03214-w)

**Uncropped images for the blots in Figure 1, Additional file 1: Fig S4-S5**

**Figure 1D: anti-Dicer**


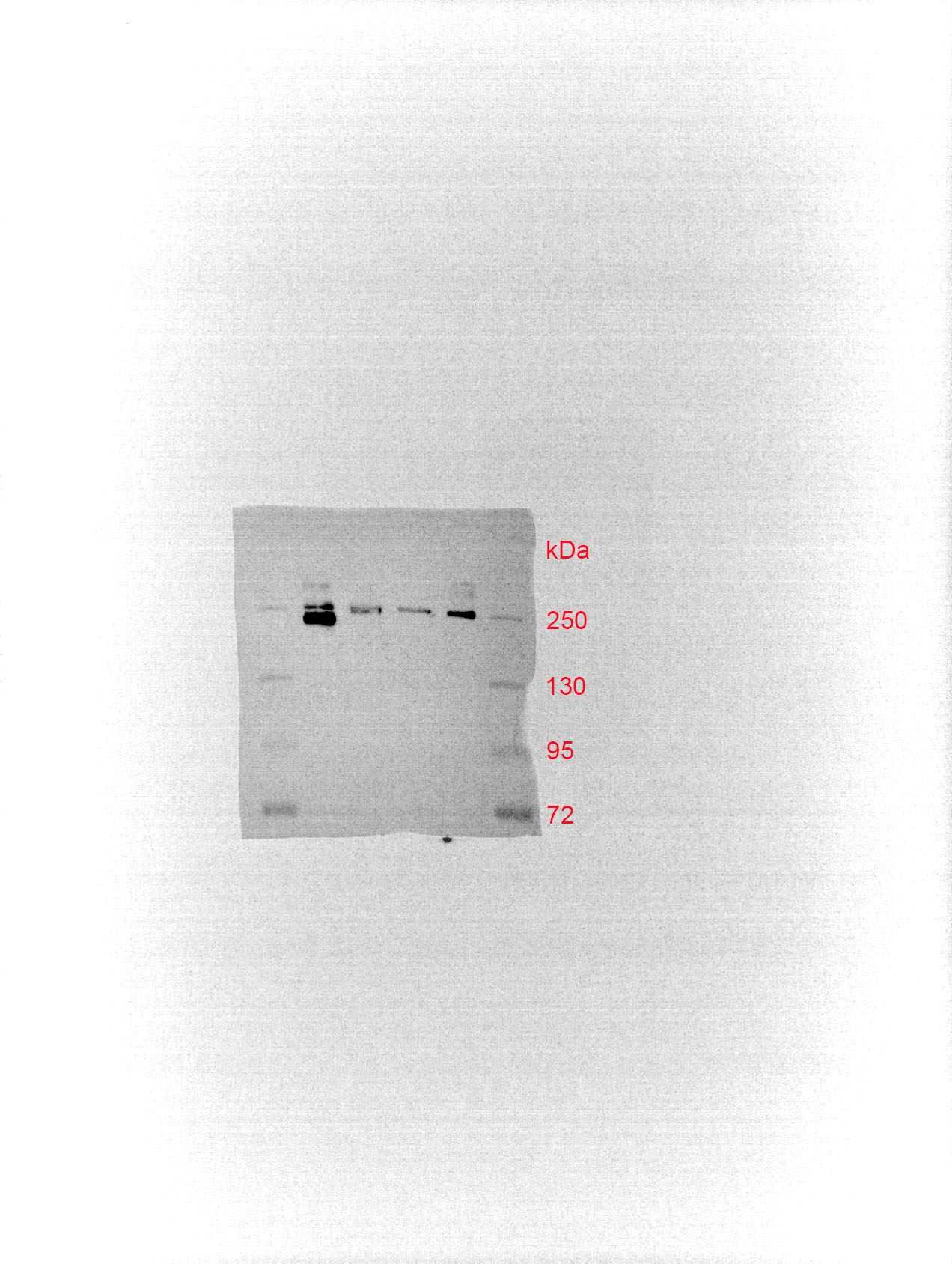


**Figure 1D: anti-Tubulin**


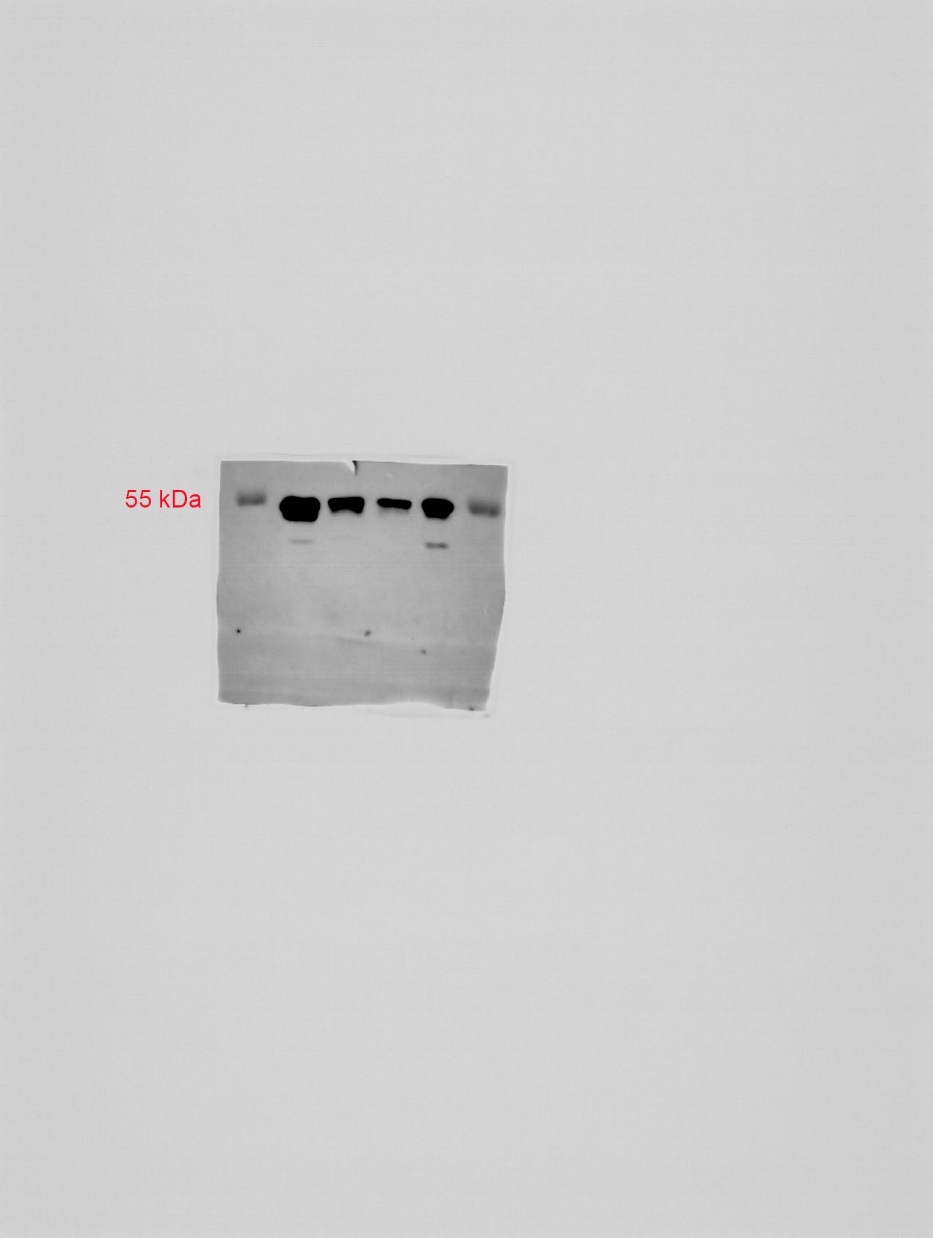


**Additional file 1: Fig S4D: GFP**


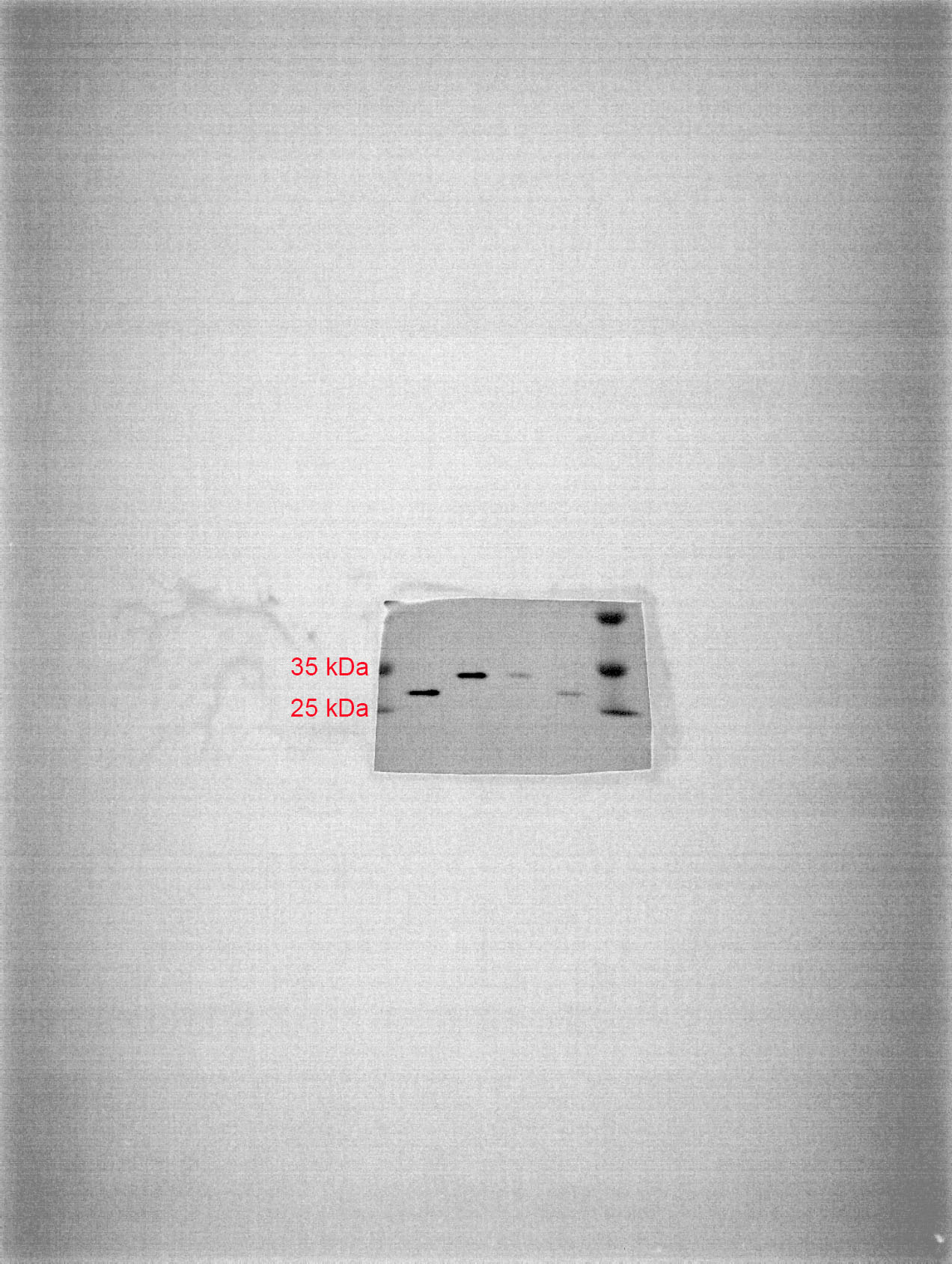


**Additional file 1: Fig S4D: co-transfected protein**


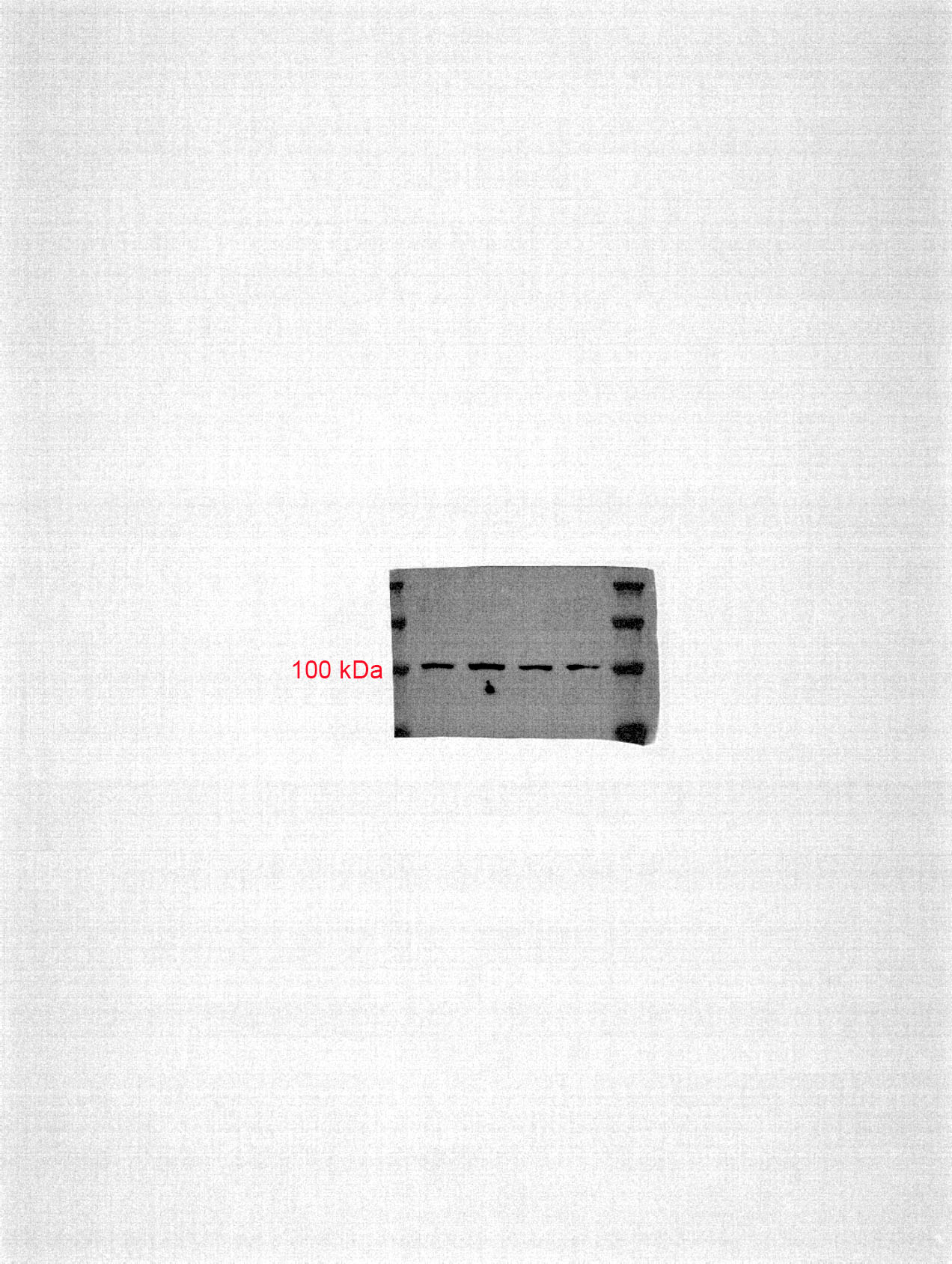


**Additional file 1: Fig S4D: Tubulin**


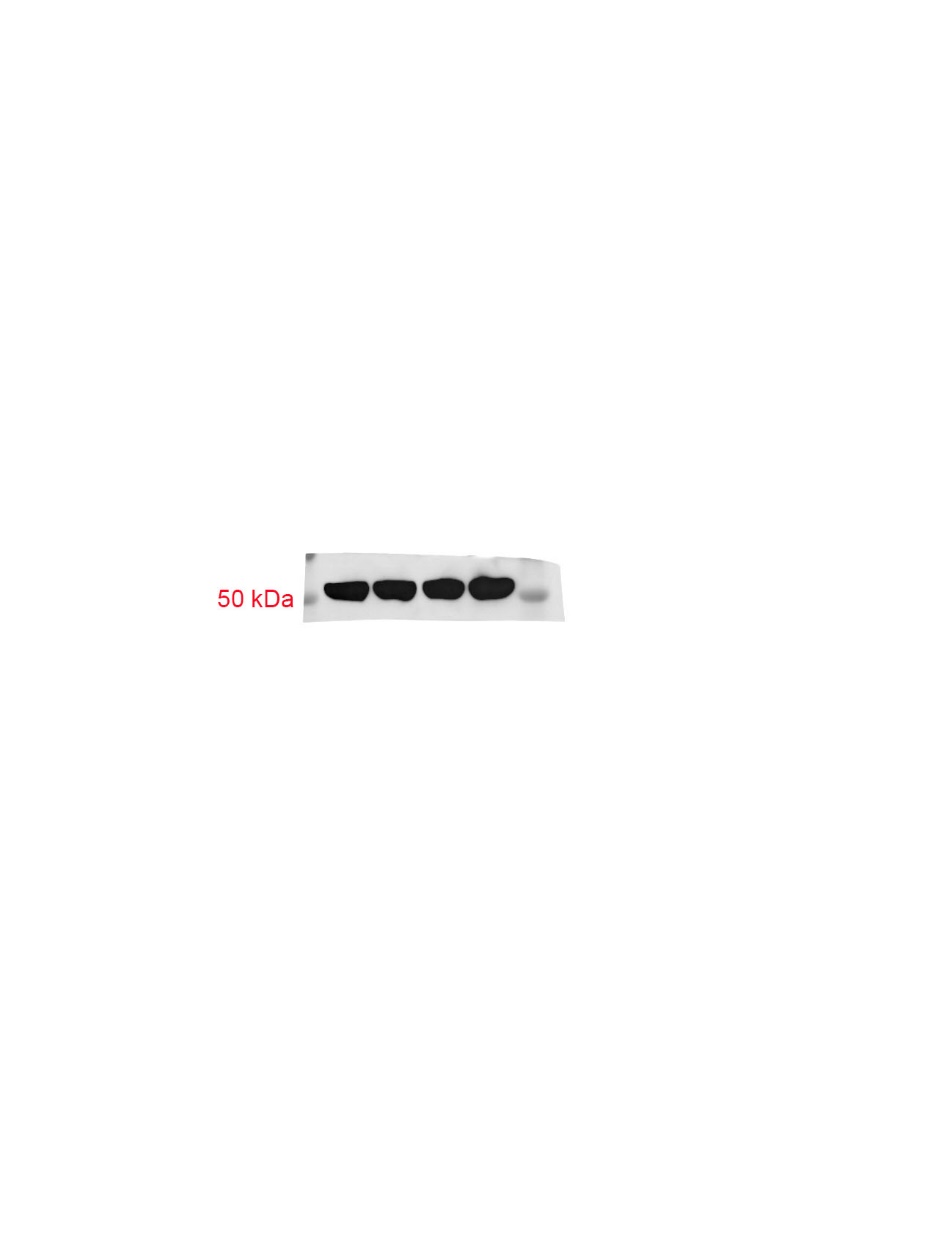


**Additional file 1: Fig S5A: Guinea pig PIWIL3**


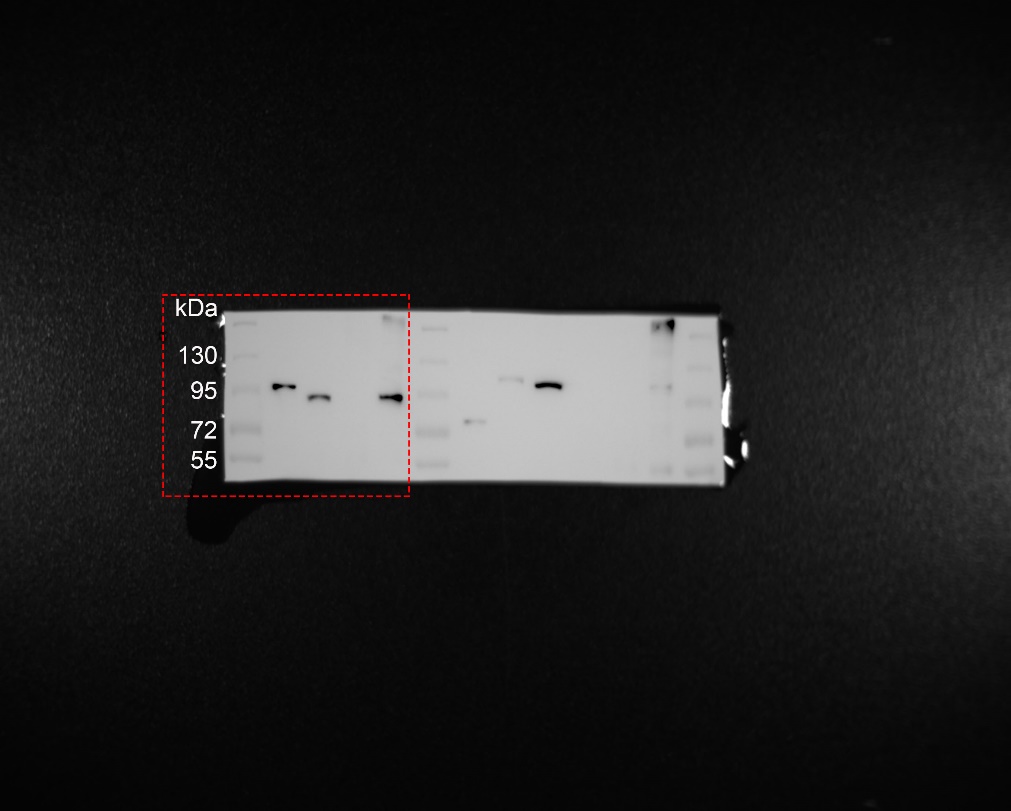


**Additional file 1: Fig S5B: Goat PIWIL3**


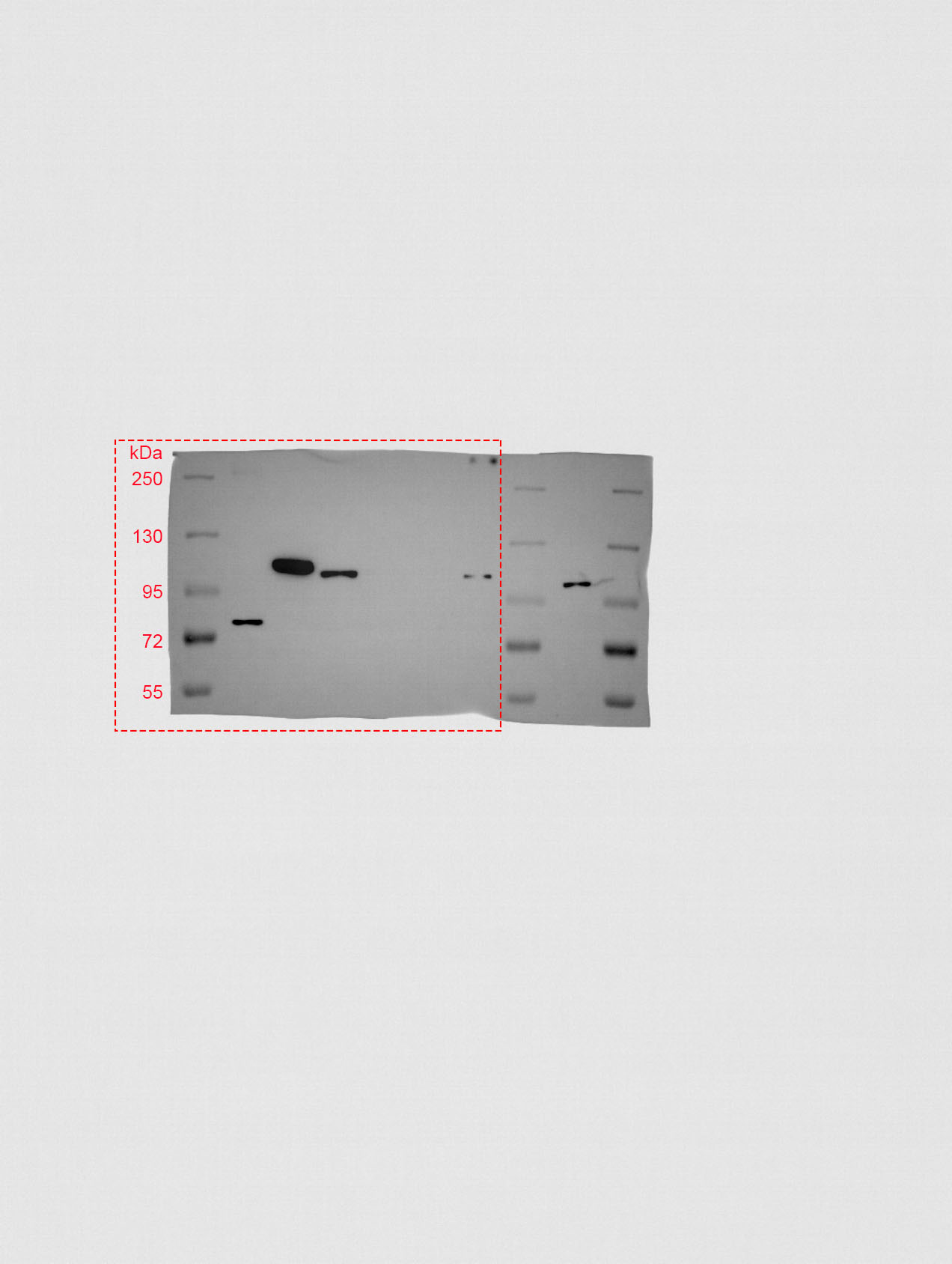


**Additional file 1: Fig S5C: Pig PIWIL3**


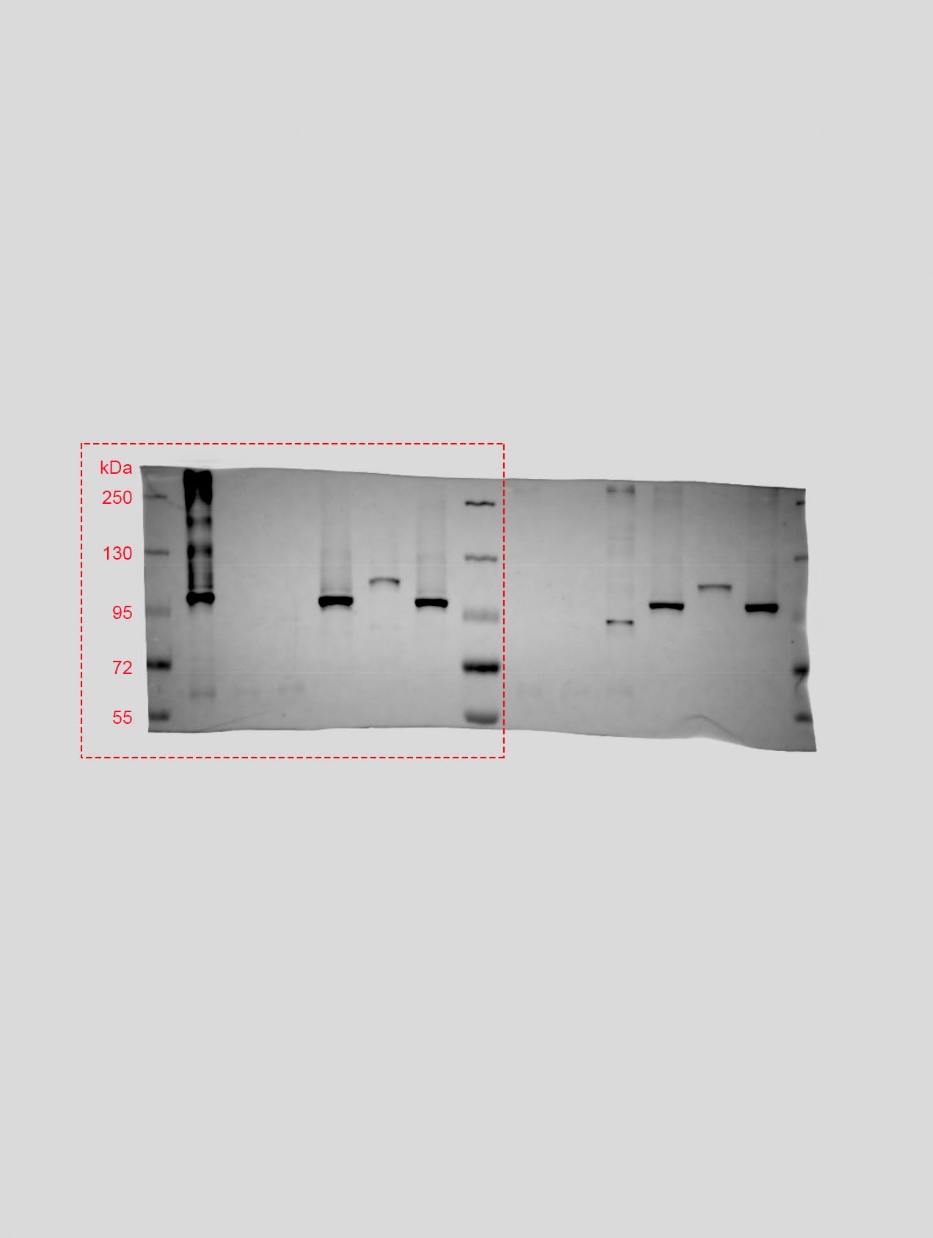


**Additional file 1: Fig S5D: Guinea pig PIWIL1**


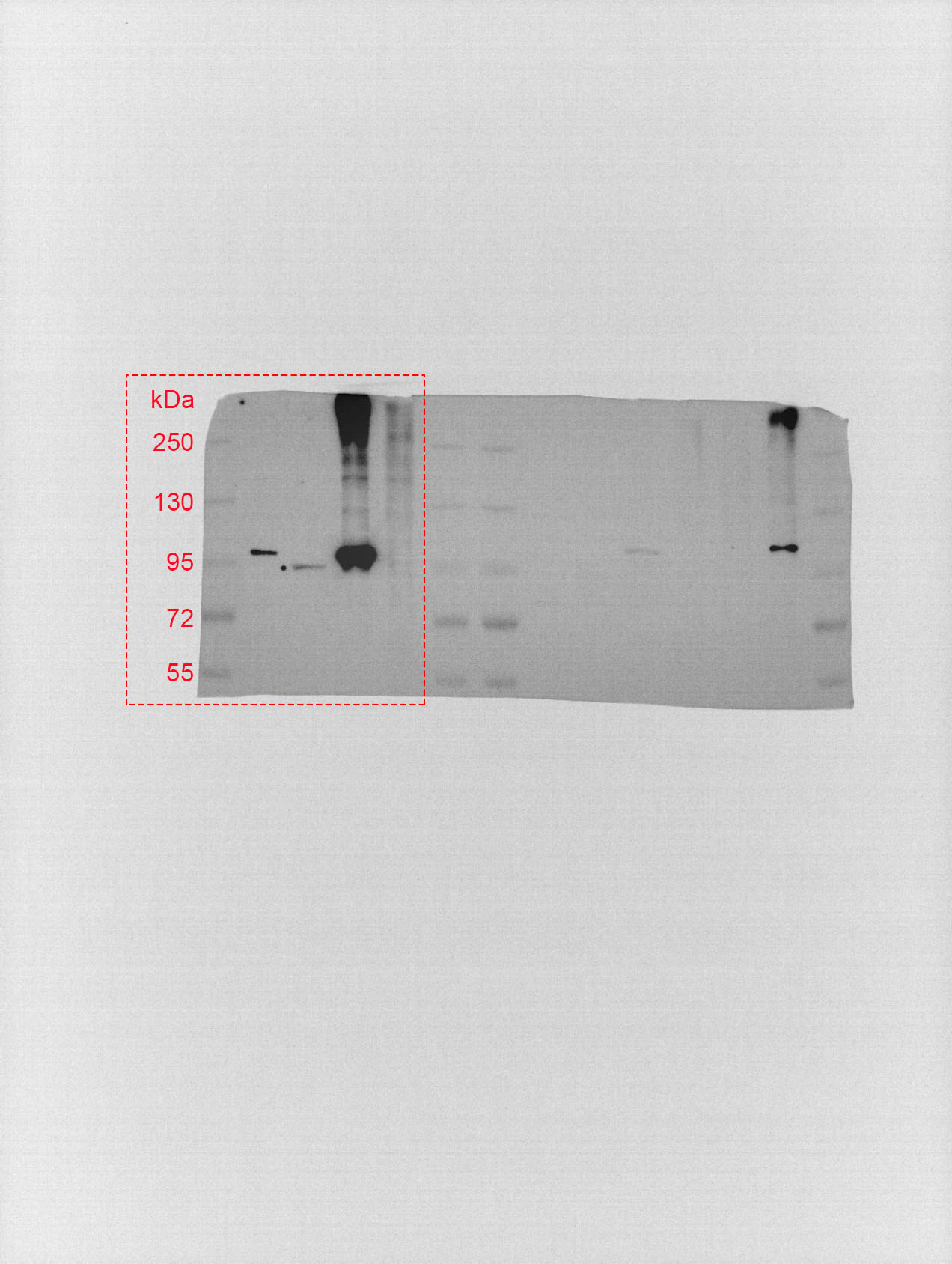


**Additional file 1: Fig S5E: Goat PIWIL1**


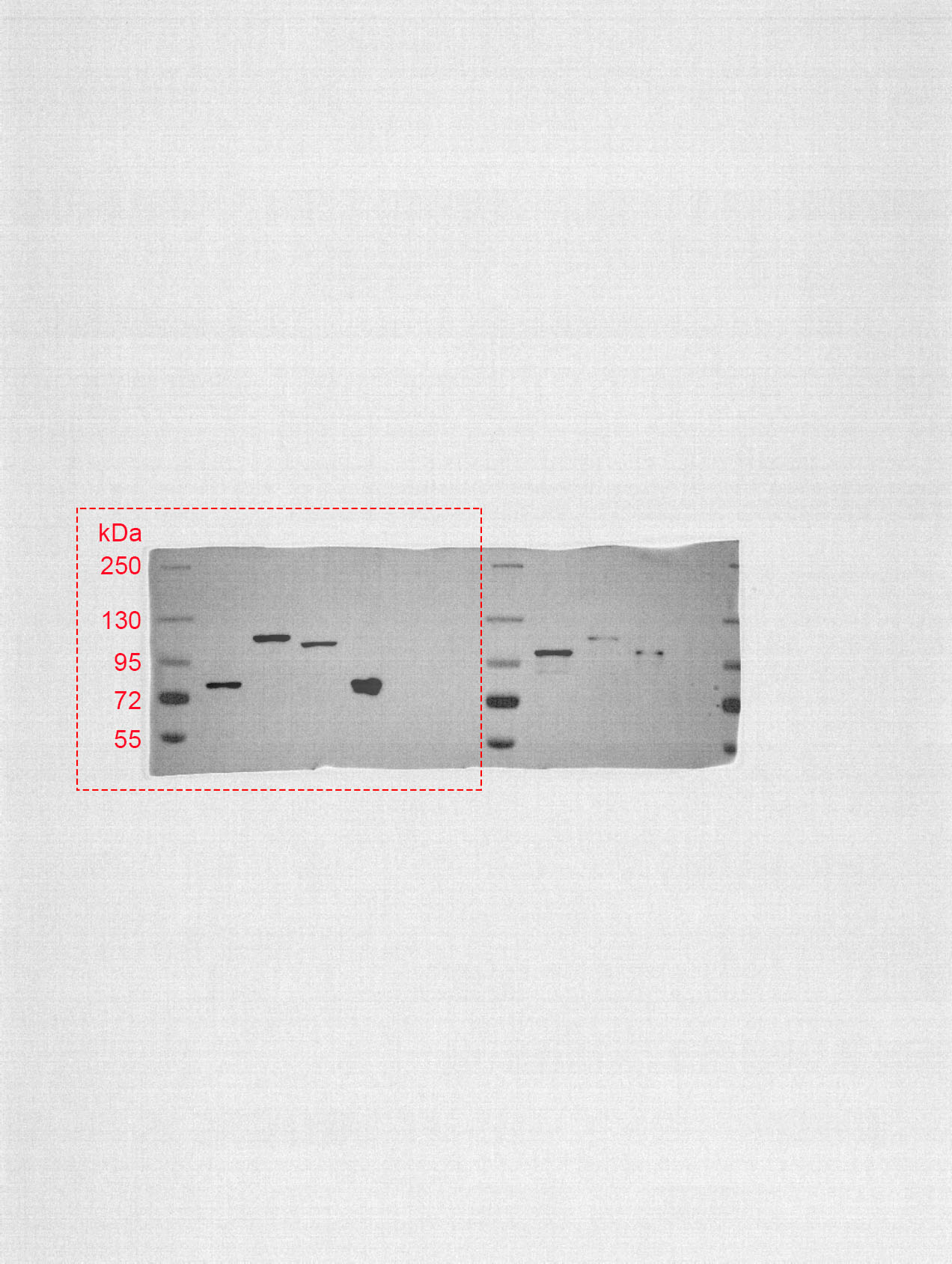


**Additional file 1: Fig S5F: Pig PIWIL1**


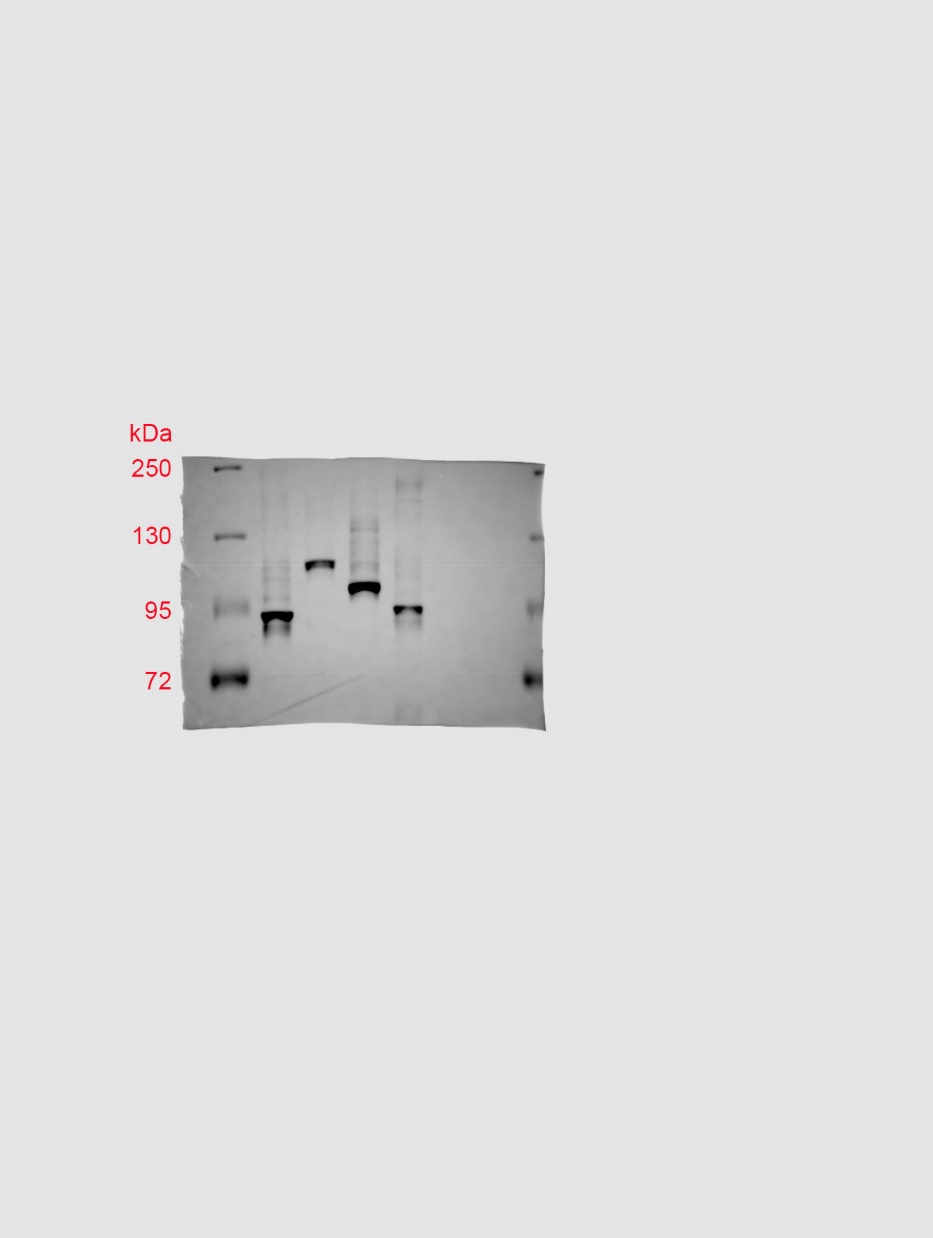


**Additional file 1: Fig S5G: Rat PIWIL1**


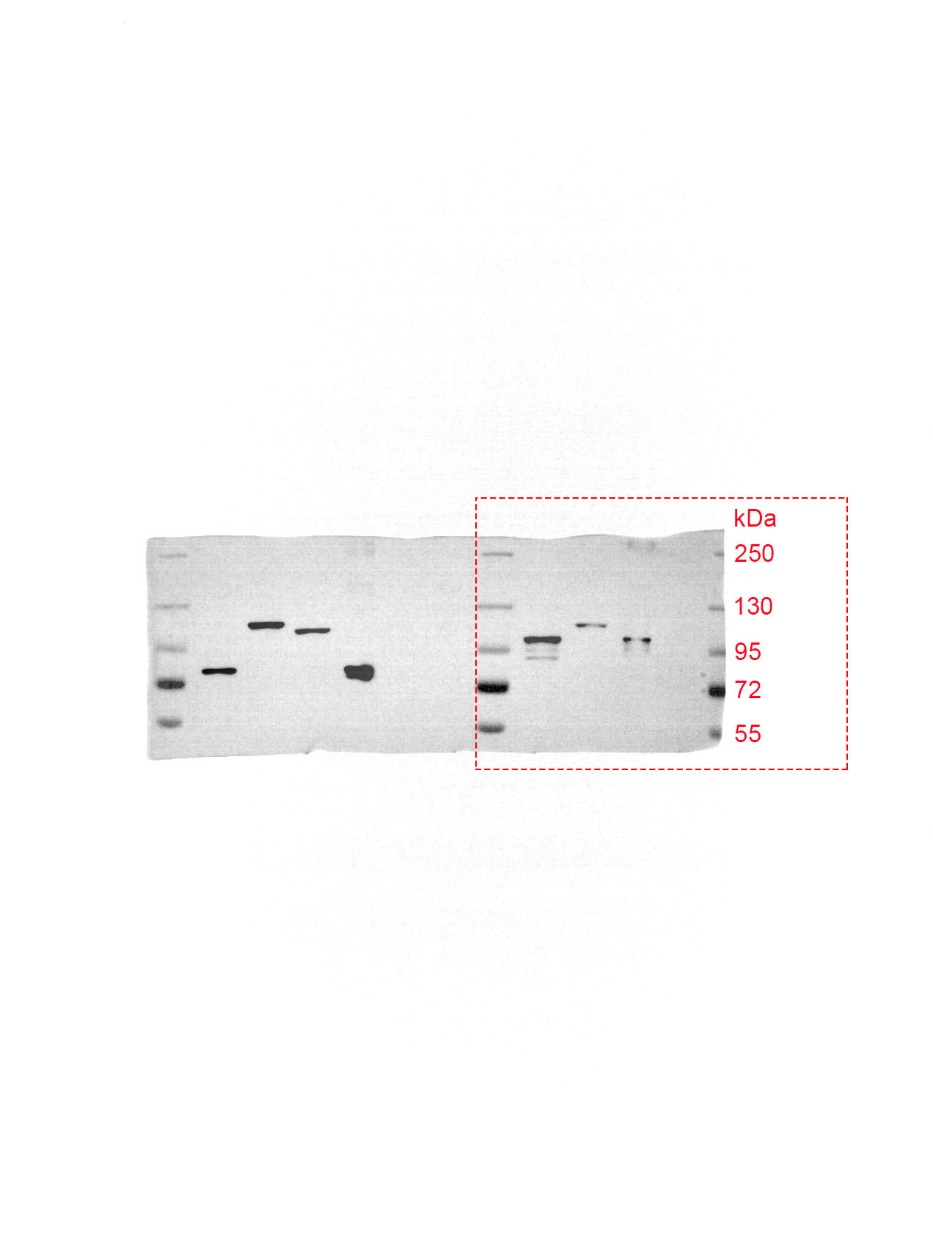

Supplement: Supplementary file 3 — Additional file 3. Uncropped images for the blots in Fig. 1, Additional file 1: Fig S4–S5. [file 13059_2024_3214_MOESM3_ESM.docx]
