# Supplementary material for: Divergent composition and transposon-silencing activity of small RNAs in mammalian oocytes
Source: Genome Biol. 2024 Mar 26;25:80. doi: 10.1186/s13059-024-03214-w (PMC10964541; doi:10.1186/s13059-024-03214-w)
Supplement: Supplementary file 4 — Additional file 4. [file 13059_2024_3214_MOESM4_ESM.docx]

**Review history**

**First round of review**

**Reviewer 1**

This paper contains important, new data on small RNAs in mammalian oocytes. In this study, the authors profile small RNAs in oocytes of 11 mammals and find that endo-siRNAs are specific to mice. They also find that small RNA composition in the oocytes is highly variable between species, potentially reflecting the specialization of small RNA functions in female germ cells. Although mice have contributed immensely to our understanding of the physiology of humans, it is also increasingly clear that the genetic and physiological differences between humans and mice hamper the extrapolation of the results obtained in mouse models to direct applications in humans. The current study also confirms this notion: mice may not be a sufficiently representative model for studying small RNA functions in oocytes. The analysis is extensive and the data are presented clearly. The novelty of the present study has been somewhat dampened by previous studies by the authors' group and others including Ketting's, Siomi's, Svoboda's and Tuschl's groups showing that mice may be rather exceptional in terms of small RNA functions in oocytes. But studies of this sort are good references and resources for further comparisons.

Some minor comments:
1. Mouse oocyte-specific Dicer (DicerO) generates endo-siRNAs in the oocyte. The gene is transcribed from a MTC element. Similar transcripts from s MTC element can be identified in rat but the transcripts do not appear to get translated into protein (DicerO). Why? Have the authors sequenced the transcripts?
2. Figure 3E shows that amounts of rat-PIWIL-associated piRNAs are faint, judged by the scale of the Y axis. This is consistent with the finding that Rat PIWIL1 expression is very low in the oocyte. Are these piRNA-like small RNAs really piRNAs?
(https://rgd.mcw.edu/rgdweb/report/gene/main.html?id=1306980)
3. In Figure 3F, all Y axes are dubbed with "spiekin," This should be "spike-in," I believe. Figure 3F shows that mouse produces small RNAs including piRNAs in the oocyte an order of magnitude higher than other mammals, judged by the scale of the Y axis. Is this correct?

**Reviewer 2**

In this study Hou, Liu and co-authors performed small RNA profiling across oocytes derived from 11 mammalian species and zebrafish. This is in part a follow up on recent observations that the piRNA pathway is essential for female fertility in golden hamsters, something that was unexpected given that the pathway is dispensable for female fertility in mice. Studying the role of small RNAs including piRNAs in oocytes of non-murine mammalian species will likely become increasingly important as previous conclusions based on mouse appears to have been an exception. In this context, the extensive data resource produced here will certainly be valuable to the field. However, I found the study a bit unfocused and it is sometimes difficult to follow what the main message is. The authors could do a better job at emphasizing what their primary hypotheses were, what results have been previously reported and what is their addition. Nevertheless, this study is valuable as the most comprehensive study of small RNAs in mammalian oocytes performed to date.

Major comments:

1. Why were oocytes collected at widely different stages? The authors previous work on hamster oocytes indicated that small RNA profiles are dynamic and change over time (PMID: 34489574), and similarly, miRNA and transposon expression is likely stage-specific. All observed differences in small RNA profiles are currently interpreted as differences between species, but I wonder if some of them are instead associated with oocyte stage? Please provide motivations for the current experimental design, perform additional experiments or controls, and/or edit the analyses or interpretations as appropriate.

2. Figure 1C: Do the authors know why the MTC element is absent from golden hamster? It is surprising given the phylogenetic relationship. Moreover, it is difficult to see what isoforms that are expressed in the current panel. I recommend using a Sashimi plot instead to also show the splice junctions.

3. Line 142: "and PCA analysis revealed a conserved pattern of miRNA expression" - What do the authors mean by this statement and how is a PCA plot supporting this? Depending on what the intended message is, the analysis may have to change.

4. Line 203: What 56 clusters are being discussed? Since 71 to 495 clusters were identified per species, there must be an additional selection criteria used here. Since large parts of the study is focused on exploring the conservation and content of these 56 piRNA clusters, it is important to clarify how they were selected.

5. Line 218: "We next compared the homology of genomic sequences of these two piRNA clusters, including their flanking genes, among species" - I am not sure I follow this analysis. First, why are flanking protein-coding genes so poorly conserved in this analysis? Second, why are piRNA cluster conservation compared with that of protein-coding genes? It may be more appropriate to compare clusters with non-coding genes or flanking intergenic regions, perhaps in terms of phyloP scores. This also connects to the claim about rapid evolution on line 227, which is not currently supported.

6. Line 559-560: "(1) more than 75% of the reads exhibited the 1U or 10A preference; and (2) at least four sequences in the cluster had the Ping-Pong signature and the ratio of Ping-Pong sequences in the cluster was > 1%" - What is the motivation for these criteria? What would be the effect of using more standard criteria (such as running proTRAC with default parameters). It is also unclear how piRNA cluster candidates were defined, did the authors use proTRAC and with what settings? Please provide a reproducible description of how piRNA clusters were defined as well as the resulting coordinates.

Minor comments:

7. Line 138: "Furthermore, the accumulation of the 10 most abundant miRNAs expression was lower than that of the corresponding 10 most abundant piRNAs" - What is the purpose of this comparison?

8. Figure 2B, 2C and 3A: The underlying data of the PCA and the clustering methods used for the heatmap should be defined. Blue-white-red is not a suitable colour scheme for continuous data since differences between blue and red look much stronger than differences between different shades of the same colour.

9. Please ensure that species terminology is correct. Guinea pig is a rodent, but is currently excluded from analyses of rodents in several places.

10. Does Fig. 4A show variability within or between species? The text indicate within, but the figure appears to show differences between species.

11. Line 276: "Intriguingly, the high levels of endo-siRNAs in oocytes of mice appears unique to this species among mammals" - This appears to be an overstatement since only 10 other mammals are tested.

12. Do the authors know why there is a 8-9 nt and 11 nt overlaps in the ping-pong analyses in Supplementary Figure S2B? Could the authors verify that no quality trimming were performed (trimming of low-quality bases at the read ends)? If performed, it would reduce both ping-pong signal and 1U/10A in a data quality-dependent manner.

13. Line 854 and 856: "Homological analysis" - I think this may not be the correct term.

**Authors’ response to reviewers**

Reviewers' comments:
Reviewer #1 (Remarks to the Author):
This paper contains important, new data on small RNAs in mammalian oocytes. In this study, the authors profile small RNAs in oocytes of 11 mammals and find that endo-siRNAs are specific to mice. They also find that small RNA composition in the oocytes is highly variable between species, potentially reflecting the specialization of small RNA functions in female germ cells. Although mice have contributed immensely to our understanding of the physiology of humans, it is also increasingly clear that the genetic and physiological differences between humans and mice hamper the extrapolation of the results obtained in mouse models to direct applications in humans. The current study also confirms this notion: mice may not be a sufficiently representative model for studying small RNA functions in oocytes. The analysis is extensive and the data are presented clearly. The novelty of the present study has been somewhat dampened by previous studies by the authors' group and others including Ketting's, Siomi's, Svoboda's and Tuschl's groups showing that mice may be rather exceptional in terms of small RNA functions in oocytes. But studies of this sort are good references and resources for further comparisons.
Response: We sincerely value the Reviewer’s time and insights. Following their suggestions, we have conducted additional experiments and analyses to address their comments, as necessary.

1. Mouse oocyte-specific Dicer (DicerO) generates endo-siRNAs in the oocyte. The gene is transcribed from a MTC element. Similar transcripts from s MTC element can be identified in rat but the transcripts do not appear to get translated into protein (DicerO). Why? Have the authors sequenced the transcripts?
Response: We again express our sincere gratitude for these insightful questions. In fact, we were also quite intrigued by this phenomenon and invested substantial efforts in unraveling differences in the specific details between animal models.
Using our RNA-seq data from mouse, rat, and golden hamster oocytes, we confirmed that the MT-C element sequence was indeed present in Dicer gene transcript (Fig. 1C). Although the MT-C elements of rat shared 84% sequence identity with that of the MT-C sequence in mouse, multiple mutations and indels were identified in the MT-C element in the rat compared with the mouse (Supplementary Fig. S4A-B). Moreover, a portion of the incorporated MTC elements occupied the full 5' untranslated region (UTR) and the alternative first exon (AltE) of DicerO transcripts. These sequence variations could potentially result in the diminished DicerO expression observed in rat oocytes compared to that in mice.
To validate this hypothesis, we constructed GFP reporter plasmids containing either the 5' UTR and the AltE from mouse or rat DicerO transcripts (see Supplementary Fig. S4C). We then transfected these plasmids into 293T cells and assessed their influence on GFP protein expression. Western blot analysis revealed that GFP fused with the mouse 5' UTR and AltE of either mouse or rat DicerO CDS was expressed at obviously higher levels than GFP fused with the rat 5’ UTR and AltE of either mouse or rat DicerO transcripts (Supplementary Figure 4D-E), suggesting that the inserted MT-C elements from rats in the 5’UTR could reduce the efficiency of DicerO translation compared to that of MT-C from mice. Although performed in vitro, this analysis provides a plausible mechanism for the absence of DicerO protein in rat oocytes. We are inclined to further speculate that additional posttranscriptional regulatory mechanisms could contribute to the almost completely abolished DicerO protein expression observed in rats in vivo. These results have been added to Supplementary Figure 4A-E (line 970) and are described in the revised Results section (lines 131-144) as follows:
“Intriguingly, Western blot data indicated that DicerO protein was the dominant isoform, compared to wild type, in mouse oocytes, but was undetectable in rat and golden hamster oocytes (Fig. 1D), suggests posttranscriptional regulation of DicerO protein expression. Notably, a portion of the incorporated MT-C elements occupied the full 5' untranslated region (UTR) and the alternative first exon (AltE) of DicerO transcripts. However, multiple mutations and indels were identified in the rat MT-C elements compared to the mouse MT-C sequence (Supplementary Fig. S4A), which shared 84% sequence identity with rat MT-C elements (Supplementary Fig. S4B). These sequence variations could potentially result in the diminished DicerO expression observed in rat oocytes compared to that in mice. We then constructed four plasmids expressing a GFP reporter fused to the 5’ UTR of DicerO transcripts and the AltE from mouse and rats, in all four possible combinations (i.e., mouse 5’ UTR plus mouse AltE; mouse 5’ UTR plus rat AltE, etc.; Supplementary Fig. S4C). GFP expression varied among the four combinations, with fusion to the rat 5’UTR resulting in the lowest expression levels (Supplementary Fig. S4D-E), suggesting that the MT-C elements inserted in the 5’UTR of rat DicerO, along with other possible factors, might reduce or abolish its expression.”
We have also added the TSS location of rat DicerO transcripts in Supplementary Fig. S4A and changed the corresponding description in the figure legend (lines 973-974):
“Multiple sequence alignment of the MT-C element from mouse and rat. Red (mouse), blue (rat), arrows indicate the TSS of DicerO transcripts.”

Supplementary Figure S4. The insertion of MT-C elements in the 5’ UTR of rat DicerO might reduce its expression
(A) Sequence alignment of the MT-C element in DicerO gene from mouse and rat genomes.
(B) Heatmap of sequence identity for the MT-C element between mouse and rat. The percent of sequence identity is shown for each comparison.
(C) A GFP reporter fused with different combinations of the MTC-containing 5’UTR and AltE of DicerO transcripts from mice or rats. CMV promoter is used to drive transgene expression in 293T cells.
(D) Western blot showing the expression of GFP protein fused with different combinations of 5’UTR and AltE from mouse or rat. The assays were independently repeated three times with similar results and a representative result is shown.
(E) Quantification of Western blots for GFP expression, normalized to Flag-PIWIL2 co-transfection, across three replicates.

2. Figure 3E shows that amounts of rat-PIWIL-associated piRNAs are faint, judged by the scale of the Y axis. This is consistent with the finding that Rat PIWIL1 expression is very low in the oocyte. Are these piRNA-like small RNAs really piRNAs?
Response: Thanks for this valuable question which has revealed a point of confusion in our data presentation. The scale of the Y-axis in Figure 3E can only be used to compare the amount of small RNAs between PIWIL immunoprecipitation and the IgG control within species. Consequently, we cannot compare the amounts of PIWI-associated piRNAs between species. We have clarified this point in the legend of Figure 3E (lines 897-899) with the sentence:
“Y-axis scale compares the amount of small RNAs between PIWIL immunoprecipitation and the IgG control within species”.

To confirm that these rat-PIWIL1-associated small RNAs are indeed piRNAs, we first verified that the expression of piRNA clusters was correlated between Rat Input and Rat PIWIL1 IP (Response Figure 1A). We then analyzed the distribution of piRNA reads of the most highly expressed piRNA cluster in rats, piC-EXD2, and found that piRNA density was highly similar between input and PIWIL1 IP for this cluster (Response Figure 1B). More importantly, piRNAs from PIWIL1-IP had strong 1U bias (Response Figure 1C), ping-pong signature (Response Figure 1D), and location in piRNA clusters on rat genome. This evidence led us to conclude that these PIWIL-associated small RNAs in rats were indeed piRNAs.

Response figure 1: Features of small RNAs immunoprecipitated by PIWIL1 from rat oocytes
(A) Correlation analysis between piRNA cluster expression levels in input and PIWIL1 IP of rat oocytes. Each point represents a piRNA cluster.
(B) PiRNA density of the most abundant piRNA cluster, piC-EXD2, in input and PIWIL1 IP in rat oocytes.
(C) Nucleotide bias in positions 1-10 of piRNAs in input and PIWIL1 IP of rat oocytes.
(D) ping-pong signatures of oocyte piRNAs in input and rat PIWIL1 IP. The Z-scores corresponding to ping-pong signatures are shown to the left of each peak.

3. In Figure 3F, all Y axes are dubbed with "spiekin," This should be "spike-in," I believe. Figure 3F shows that mouse produces small RNAs including piRNAs in the oocyte an order of magnitude higher than other mammals, judged by the scale of the Y axis. Is this correct?
Response: We appreciate the reviewer for bringing this mistake to our attention, which we have corrected in revised Figure 3F.
Due to the high variation in total small RNA amounts between oocytes of different species, we introduced varying quantities of spike-in into oocyte samples of different species before NaIO4 oxidation treatment. Thus, the Y-axis scales were specifically used to compare the levels of small RNAs with and without NaIO4 treatment within each species, but not between species. We regret any confusion arising from this mistake. We have provided clarification in the legend of Figure 3F (lines 901-902), as follows:
“Y-axis scales are used for within-species comparisons of small RNA levels with or without NaIO4 treatment.”

We also provide further clarification in the legend of Figure 3B-D (lines 895-896), as follows:
“The Y-axis scale compares the amount of small RNAs between PIWIL immunoprecipitation and IgG controls within species.”

Reviewer #2 (Remarks to the Author):
In this study Hou, Liu and co-authors performed small RNA profiling across oocytes derived from 11 mammalian species and zebrafish. This is in part a follow up on recent observations that the piRNA pathway is essential for female fertility in golden hamsters, something that was unexpected given that the pathway is dispensable for female fertility in mice. Studying the role of small RNAs including piRNAs in oocytes of non-murine mammalian species will likely become increasingly important as previous conclusions based on mouse appears to have been an exception. In this context, the extensive data resource produced here will certainly be valuable to the field. However, I found the study a bit unfocused and it is sometimes difficult to follow what the main message is. The authors could do a better job at emphasizing what their primary hypotheses were, what results have been previously reported and what is their addition. Nevertheless, this study is valuable as the most comprehensive study of small RNAs in mammalian oocytes performed to date.
Response: We genuinely appreciate the Reviewer’s time and insightful comments. Previous studies have already highlighted the distinct small RNA expression between human and mouse oocytes and emphasized the unique patterns of PIWIL3-piRNA distribution between human and golden hamster, but not mouse, oocytes. In our current work, we aimed to explore and compare small RNA composition across a broader range of mammalian oocytes, which has not been extensively reported. Following the Reviewer’s advice, we have conducted additional experiments, performed more thorough analyses, and revised the manuscript to focus our message and explain our hypotheses.

1. Why were oocytes collected at widely different stages? The authors previous work on hamster oocytes indicated that small RNA profiles are dynamic and change over time (PMID: 34489574), and similarly, miRNA and transposon expression is likely stage-specific. All observed differences in small RNA profiles are currently interpreted as differences between species, but I wonder if some of them are instead associated with oocyte stage? Please provide motivations for the current experimental design, perform additional experiments or controls, and/or edit the analyses or interpretations as appropriate.
Response: We thank the reviewer for this insightful question, and we acknowledge the potential limitations of comparing oocytes at different stages.
Initially, our goal was to gather MII oocytes from different species at the same developmental stage for subsequent analysis and comparison. However, it is worth noting that many species included in our study are not conventional model organisms, posing great technical challenges for the collection of mature oocytes. Among these species, the secondary follicle of the dog differs considerably from the GV or MII oocytes of the other 11 species. With the assistance of a veterinary expert, we re-collected dog MII oocytes and performed small RNA and mRNA sequencing. The updated results have been incorporated into our revised manuscript, including Figure1B-C (line 861), Figure 2 (line 880), Figure 3A, 3F (line 888), Figure 4 (line 906), Figure 5 (line 932), Supplementary Figure 1 (line 946), Supplementary Figure 2 (line 952), Supplementary Figure 3 (line 958), Supplementary Figure 7 (line 1003), Supplementary Figure 8 (line 1015), Supplementary Figure 9 (line 1033), Supplementary Figure 10 (line 1040), Supplementary Figure 11B (line 1047), Supplementary Figure 12 (line 1055).

We have changed our description in line 94 as follows:
“All oocytes were collected at the germinal vesicle (GV) or MII stage”, and in the Supplementary Figure1 legend (line 949).

We have changed the Methods section (lines 412-417) as follows:
“MII oocytes were collected from 12- to 24-month-old beagles on the estimated ovulation day through continuous observation. The collected oocytes were transferred into M199 medium containing 25 mM HEPES. Cumulus cells were removed by adding hyaluronidase (2 mg/ml) with gentle pipetting. The oocytes were then washed twice in DPBS and each oocyte was transferred to a separate PCR tube containing < 0.5 μl DPBS and stored at -80 °C until use”.

In addition, we have added some study limitations arising from small RNA expression profiling at different oocyte stages, especially in different species, to the revised Discussion section (lines 339-343):
“However, some limitations should be considered when interpreting our results. In particular, not all oocytes collected from the 12 species reached the MII stage, when oocytes are most functional. Since the expression of miRNAs and TEs are likely stage-specific, more precise comparisons are warranted in future work using oocytes of the same stage between species.”

2. Figure 1C: Do the authors know why the MTC element is absent from golden hamster? It is surprising given the phylogenetic relationship. Moreover, it is difficult to see what isoforms that are expressed in the current panel. I recommend using a Sashimi plot instead to also show the splice junctions.
Response: We thank the reviewer for this constructive suggestion. We found very few (i.e., almost undetectable) DicerO transcripts driven by the MT-C element in the golden hamster, which may be due to functional sequence variations that arose in the MT-C promoter during evolution. As suggested, we generated a Sashimi plot to compare differences in the splice junctions of DicerO between mice, rats and golden hamsters. These plots reveal the notably high expression of MTC-containing isoforms in mice and rats, but not golden hamsters. The updated results have been added to Figure 1C (line 861) of the revised manuscript.

The Figure 1C legend (lines 869-873) now reads:
“Sashimi plot of splice junctions in Dicer expressed in oocytes of mouse, rat, and golden hamster. The red asterisk indicates the RNA-seq signal of the MT-C element. IGV was used to compute the junction track from alignment data. Junctions from the + strand are colored red and extend above the center line. Junctions from the – strand are blue and extend below the center line. Arc height and thickness are proportional to the depth of read coverage.”


Figure 1C. Sashimi plot of splice junctions in Dicer expressed in oocytes of mouse, rat, and golden hamster.
The red asterisk indicates the RNA-seq signal of the MT-C element. IGV was used to compute the junction track from alignment data. Junctions from the + strand are colored red and extend above the center line. Junctions from the – strand are blue and extend below the center line. Arc height and thickness are proportional to the depth of read coverage.

3. Line 142: "and PCA analysis revealed a conserved pattern of miRNA expression" - What do the authors mean by this statement and how is a PCA plot supporting this? Depending on what the intended message is, the analysis may have to change.
Response: We appreciate the reviewer's concern, and we understand that PCA plots cannot support “a conserved pattern of miRNA expression”. We have revised the statement accordingly to reflect more accurately reflect our findings (lines 156-158):
"Principal component analysis (PCA) showed obvious clustering of samples from the same species, but little overlap between species, suggesting that overall miRNA profiles are distinct among these 12 species."

We also deleted “as well as the patterns of miRNA expression” in lines 168 and “patterns” in line 297.

In addition, a heatmap of the 100 most abundant miRNA families in oocytes showed little difference among species after re-performed MII oocyte collection from dogs. We therefore modified our description of these results (line 158-160) as follows:
“Notably, most of the highly expressed miRNAs, including miR-125-5p/351-5p, miR-99-5p/100-5p, miR-30-5p, miR-2478, and let-7-5p/98-5p, were conserved among the 12 vertebrates.”

As well as the description in line 163:
“miR-290/292-5p/293-5p, miR-292a-3p/467a-5p, which were uniquely detected in rat oocytes”.

4. Line 203: What 56 clusters are being discussed? Since 71 to 495 clusters were identified per species, there must be an additional selection criteria used here. Since large parts of the study is focused on exploring the conservation and content of these 56 piRNA clusters, it is important to clarify how they were selected.
Response: We selected these 56 piRNA clusters based on their higher expression in either most (i.e., 9 or more) of the 12 species, or in only a few (i.e., 1-3) individual or closely related species. These criteria have been revised in the Results section (lines 218-220) as follows:
“To explore the evolution of piRNA clusters among species, we performed synteny analysis on a subset of piRNA clusters that were highly expressed in either most (i.e., 9 or more) of the 12 species or in only individual or a few closely related species.”

We also provided the location of the piRNA clusters identified in each species in Supplementary Table 6 and changed the description in line 214.

5. Line 218: "We next compared the homology of genomic sequences of these two piRNA clusters, including their flanking genes, among species" - I am not sure I follow this analysis. First, why are flanking protein-coding genes so poorly conserved in this analysis? Second, why are piRNA cluster conservation compared with that of protein-coding genes? It may be more appropriate to compare clusters with non-coding genes or flanking intergenic regions, perhaps in terms of phyloP scores. This also connects to the claim about rapid evolution on line 227, which is not currently supported.
Response: We thank the reviewer for this important comment. The flanking protein-coding genes are poorly conserved because we used the genomic sequences of genes (i.e., including introns) for sequence alignment. Following the reviewer’s suggestion, we calculated phyloP scores for the piRNA clusters, flanking intergenic regions (including 10 kb upstream and downstream of the cluster), and cDNA sequences of flanking protein-coding genes (served as positive control) (Figure 4C, Supplementary Figure S8B). Interestingly, we found that the phyloP scores of the piRNA cluster were significantly lower than that of the flanking intergenic region and cDNA sequencing of adjacent protein-coding genes, which suggested that the sequences in piRNA clusters were evolving more rapidly than their respective flanking regions. We have incorporated these results into the revised manuscript (Figure 4C and Supplementary Figure S8B) and modified our description in lines 234-240 as follows:
“In order to compare conservation in the homology of genomic sequences of these two piRNA clusters and their flanking regions among species, we calculated phyloP scores for the piRNA clusters, the 10 kb upstream and downstream flanking intergenic regions, and the cDNA sequences of flanking protein-coding genes. Interestingly, the piRNA clusters had significantly lower phyloP scores than the flanking intergenic regions as well as the cDNA sequences of adjacent protein-coding genes, which suggested that these piRNA clusters were evolving more rapidly than both the flanking protein-coding and non-coding genomic regions.”

We have provided clarification of this analysis in the legend of Figure 4C (lines 915-920):
“The phyloP scores of the piC-ZNF518B-WDR1 of piRNA cluster, its 10 kb upstream and downstream flanking intergenic regions and cDNA sequences of adjacent protein-coding genes. The values represent -log p-values under a null hypothesis of neutral evolution. The sites predicted to be conserved are assigned positive scores while sites predicted to undergo accelerated evolution are assigned negative scores.”

And in the Supplementary Figure S8B legend (lines 1019-1022):
“The phyloP scores of piRNA cluster piC-RBM19-TBX5, its flanking intergenic region, and cDNA sequences of its flanking protein-coding genes. The values represent -log p-values under a null hypothesis of neutral evolution. The sites predicted to be conserved are assigned positive scores while sites predicted to undergo accelerated evolution are assigned negative scores.”

We also expanded our explanation of PhyloP score calculation in the revised Methods (lines 637-642):
“We used the likelihood ratio test (LRT) method and CONACC mode from phyloP to compute conservation scores for each site in the alignment. We then divided the site equally into 100 bins according to their coordinates and calculated average phyloP scores for each bin. The values represent -log p-values under a null hypothesis of neutral evolution. The sites predicted to be conserved are assigned positive scores while sites predicted to undergo accelerated evolution are assigned negative scores.”

We have deleted the description of Multiple sequence alignment in the Methods (lines 632-635).


Figure 4C. The phyloP scores of the piC-ZNF518B-WDR1 of piRNA cluster, its 10 kb upstream and downstream flanking intergenic regions and cDNA sequences of adjacent protein-coding genes. The values represent -log p-values under a null hypothesis of neutral evolution. The sites predicted to be conserved are assigned positive scores while sites predicted to undergo accelerated evolution are assigned negative scores.
Supplementary Figure S8B. The phyloP scores of piRNA cluster piC-RBM19-TBX5, its flanking intergenic region, and cDNA sequences of its flanking protein-coding genes. The values represent -log p-values under a null hypothesis of neutral evolution. The sites predicted to be conserved are assigned positive scores while sites predicted to undergo accelerated evolution are assigned negative scores.

6. Line 559-560: "(1) more than 75% of the reads exhibited the 1U or 10A preference; and (2) at least four sequences in the cluster had the Ping-Pong signature and the ratio of Ping-Pong sequences in the cluster was > 1%" - What is the motivation for these criteria? What would be the effect of using more standard criteria (such as running proTRAC with default parameters). It is also unclear how piRNA cluster candidates were defined, did the authors use proTRAC and with what settings? Please provide a reproducible description of how piRNA clusters were defined as well as the resulting coordinates.
Response: We thank the reviewer for this constructive advice. ProTRAC (v2.4.2) was indeed used to detect piRNA clusters, as referenced in the manuscript. To minimize potential noise, we implemented stringent parameters for identifying piRNA clusters across the 12 species (-1Tor10A 0.75 -1Tand 10A 0.5). To accommodate variation in the distribution of small RNA sequence lengths among these species, we set the piRNA length limit to 17-23nt (-pimin 17 -pimax 23) in rabbits and guinea pigs. For mice, rats, Chinese hamsters, and zebrafish, the piRNA length limit was set to 24-32nt (-pimin 24 -pimax 32). In humans, monkeys, golden hamsters, dogs, goats, and pigs, the piRNA length limit was set to 17-32nt (-pimin 17 -pimax 32). Other parameters of proTRAC were maintained at default parameters. We have expanded our description of the method for defining piRNAs and piRNA clusters in the revised Methods section (lines 592-598) as follows:
“To remove potential noise, we used stringent parameters in proTRAC (v2.4.2) to identify piRNA clusters in these 12 species (-1Tor10A 0.75 -1Tand10A 0.5). Due to differences in the length distribution of small RNA sequences in these species, we set the piRNA length limit to 17-23nt (-pimin 17 -pimax 23) in rabbits and guinea pigs. In mice, rats, Chinese hamsters and zebrafish we set the piRNA length limit to 24-32nt (-pimin 24 -pimax 32). In humans, monkeys, golden hamsters, dogs, goats, and pigs, we set the piRNA length limit to 17-32nt (-pimin 17 -pimax 32). Other parameters in proTRAC were not changed, and the default parameters were used.”

The resulting coordinates of piRNA clusters are now provided in Supplementary Table S6.

We have also improved the description of our method for defining endo-siRNAs and endo-siRNA clusters in the revised Methods (lines 605-608):
“We used proTRAC (v2.4.2) to identify endo-siRNA clusters in mouse oocytes (-pimin 17 -pimax 24 -1Tor10A 0.25 -1Tand10A 0.1 -pdens 0.01 -clstrand 0.5)”. The resulting coordinates of endo-siRNA clusters are listed in Supplementary Table S6.

7. Line 138: "Furthermore, the accumulation of the 10 most abundant miRNAs expression was lower than that of the corresponding 10 most abundant piRNAs" - What is the purpose of this comparison?
Response: We originally aimed to highlight the low expression of miRNAs in these 12 species. However, upon further consideration, we realized that conducting this comparison did not contribute meaningfully to our study. We have deleted this analysis in the revised manuscript (line 153-154).

8. Figure 2B, 2C and 3A: The underlying data of the PCA and the clustering methods used for the heatmap should be defined. Blue-white-red is not a suitable colour scheme for continuous data since differences between blue and red look much stronger than differences between different shades of the same colour.
Response: We appreciate this valuable advice regarding data presentation. We have added details of the underlying data used for PCA and hierarchical clustering in the Figure 2B legend (lines 883-884): “total miRNA families were used for PCA analysis”, the legend of Figure 2C in lines 885-886: “Hierarchical clustering was used to generate a heatmap (method=‘complete’)”, the figure legend of 3A (line 892): “Hierarchical clustering was used to generate a heatmap (method=‘complete’)”, and the Figure S3 legend (line 962): “Hierarchical clustering was used to generate a heatmap (method=‘complete’)”.

We have also changed the color scheme of heatmaps in Figure 2C, Figure 3A, and Figure S3 of the revised manuscript.

9. Please ensure that species terminology is correct. Guinea pig is a rodent, but is currently excluded from analyses of rodents in several places.
Response: We thank the reviewer for pointing out this mistake. We have added guinea pig to our analysis in Figure S6A (line 993) and changed the description in the figure legend (lines 995-996) as follows: “Comparison of piRNA and endo-siRNA expression in five rodent species: mouse, rat, Chinese hamster, golden hamster, and guinea pig.”

We also modified our description in line 203-204 as follows:
“In the five rodents, only golden hamsters and guinea pigs expressed Piwil3 and os-piRNAs”.

10. Does Fig. 4A show variability within or between species? The text indicate within, but the figure appears to show differences between species.
Response: We thank the reviewer for pointing out this mistake. We have changed “within” to “between” in the renewed manuscript (line 225).

11. Line 276: "Intriguingly, the high levels of endo-siRNAs in oocytes of mice appears unique to this species among mammals" - This appears to be an overstatement since only 10 other mammals are tested.
Response: We thank the reviewer for pointing out this unintentional overstatement. Lines 298-300 now read: “Intriguingly, the high levels of endo-siRNAs observed in mouse oocytes appear unique among the 11 mammals studied here, suggesting the possibility that mammals might express endo-siRNAs at generally very low levels, with mice representing an exception.”

12. Do the authors know why there is a 8-9 nt and 11 nt overlaps in the ping-pong analyses in Supplementary Figure S2B? Could the authors verify that no quality trimming were performed (trimming of low-quality bases at the read ends)? If performed, it would reduce both ping-pong signal and 1U/10A in a data quality-dependent manner.
Response: We appreciate the reviewer’s suggestion. In our previous analysis, we used cutadapt to eliminate low-quality bases and 3' adapter sequences in small RNAs, ensuring the removal of low-quality bases. Following the reviewer’s suggestion, we revisited our data and recognized that all piRNAs were used for ping-pong signal calculation in our initial analysis, which could introduce significant noise to the ping-pong signal. It has been well established that the ping-pong signal reflects the piRNA characteristic of actively cutting TE transcripts, which not all piRNAs exhibit. Therefore, we have refined our approach by specifically using only TE-derived piRNAs for ping-pong signal calculation. The updated results reveal a more pronounced 10 nt overlapping ping-pong signal. This new result is presented in revised Figure S2B (line 952).

13. Line 854 and 856: "Homological analysis" - I think this may not be the correct term.
Response: We thank the reviewer for pointing out this mistake. We have removed it and changed our description of the phyloP plot of evolutionary rates in the revised manuscript (lines 915-920).

**Second round of review**

**Reviewer 1**

I will not describe the main achievements of the paper, since this was done in the review of the original submission. On the whole it has been significantly improved. I want to thank and commend the authors for actually experimentally addressing all the reviewers' concerns. The result is a clear and strong manuscript!

**Reviewer 2**

The authors have thoroughly addressed all of my comments. In particular, they have re-collected dog MII oocytes, making these data more comparable to other species and improved their analyses and descriptions thereof in several places.

The study will be valuable for the field and I recommend it for publication.
